# Glycation Interferes with the Activity of the Bi-Functional UDP-*N*-Acetylglucosamine 2-Epimerase/*N*-Acetyl-mannosamine Kinase (GNE)

**DOI:** 10.3390/biom13030422

**Published:** 2023-02-23

**Authors:** Vanessa Hagenhaus, Jacob L. Gorenflos López, Rebecca Rosenstengel, Carolin Neu, Christian P. R. Hackenberger, Arif Celik, Klara Weinert, Mai-Binh Nguyen, Kaya Bork, Rüdiger Horstkorte, Astrid Gesper

**Affiliations:** 1 Institute for Physiological Chemistry, Medical Faculty, Martin-Luther-University Halle-Wittenberg, 06114 Halle, Germany; 2 Leibniz-Forschungsinstitut für Molekulare Pharmakologie im Forschungsverbund Berlin e.V. (FMP), Campus Berlin-Buch, Robert-Roessle-Str. 10, 13125 Berlin, Germany; 3 Institut für Chemie, Humboldt Universität zu Berlin, Brook-Taylor-Str. 2, 12489 Berlin, Germany

**Keywords:** GNE-myopathy, glycation, methylglyoxal, sialylation, post-translational modification, adult-onset disease

## Abstract

Mutations in the gene coding for the bi-functional UDP-*N*-acetylglucosamine 2-epimerase/*N*-acetylmannosamine kinase (GNE), the key enzyme of the sialic acid biosynthesis, are responsible for autosomal-recessive GNE myopathy (GNEM). GNEM is an adult-onset disease with a yet unknown exact pathophysiology. Since the protein appears to work adequately for a certain period of time even though the mutation is already present, other effects appear to influence the onset and progression of the disease. In this study, we want to investigate whether the late onset of GNEM is based on an age-related effect, e.g., the accumulation of post-translational modifications (PTMs). Furthermore, we also want to investigate what effect on the enzyme activity such an accumulation would have. We will particularly focus on glycation, which is a PTM through non-enzymatic reactions between the carbonyl groups (e.g., of methylglyoxal (MGO) or glyoxal (GO)) with amino groups of proteins or other biomolecules. It is already known that the levels of both MGO and GO increase with age. For our investigations, we express each domain of the GNE separately, treat them with one of the glycation agents, and determine their activity. We demonstrate that the enzymatic activity of the *N*-acetylmannosamine kinase (GNE-kinase domain) decreases dramatically after glycation with MGO or GO—with a remaining activity of 13% ± 5% (5 mM MGO) and 22% ± 4% (5 mM GO). Whereas the activity of the UDP-*N*-acetylglucosamine 2-epimerase (GNE-epimerase domain) is only slightly reduced after glycation—with a remaining activity of 60% ± 8% (5 mM MGO) and 63% ± 5% (5 mM GO).

## 1. Introduction

GNE myopathy (GNEM; OMIM 605820) is an autosomal-recessive disease, caused by mutations in the gene encoding the bi-functional UDP-*N*-acetylglucosamine 2-epimerase/*N*-acetylmannosamine kinase (GNE), which is the key enzyme of the sialic acid biosynthesis [1,2]. Sialic acids have a plethora of functions; they are important for cellular and molecular recognition [3], they play a role in cell adhesion [4,5] and migration [4,6], and they can be involved in the transport of charged molecules [3]. Until now, more than 200 mutations have been known which lead to GNEM [7,8]. Beside them are certain so-called founder mutations, e.g., M743T (Note: In the original paper the mutation was named M712T [9]. Nevertheless, a change in the nomenclature of GNE-variants led to a rename into M743T [10].), which is a founder mutation in the Middle Eastern population [9], or V603L, which is a founder mutation in Japanese individuals [11,12]. 

Another genetically related disease that is also based on mutations in GNE is sialuria (OMIM 269921) [13,14,15]. GNEM is associated with hyposialylation [16,17,18]—but it should be noted here, however, that the connection between (hypo)sialylation and GNEM has not yet been finally clarified [19,20]. 

The *GNE* gene consists of 14 exons, exon A1 and exons 1 to 13 [21]. So far, nine different mRNA splice variants are known, leading to the six already found GNE isoforms 1-6 (two transcripts, NM_001190388.2 and NM_001374798.1, are leading to isoform 3 [22]), and the two predicted isoforms X1 and X2 [23,24]. Furthermore, GNE and its different isoforms are unevenly expressed in different tissues, with high RNA expression in the liver and low RNA expression in skeletal muscle [23,25,26,27]. 

The two domains of the GNE—the epimerase (E.C. 5.1.3.14; [28,29,30]) and the kinase (E.C. 2.7.1.60; [31,32,33])—are linked and act as a bi-functional enzyme [34,35]. Nevertheless, they also show activities, albeit lower than the wild-type enzyme, when considered individually [36]. One benefit of a bi-functional enzyme with two domains at once is the possibility for substrate channeling, as for example is already known for the formiminotransferase cyclodeaminase (FTCD) [37] or the dihydrofolate reductase-thymidylate synthase [38]. Overall, there are different ways of transferring the substrate between the two active centers of an enzyme, e.g., through a kind of tunnel in the enzyme [39] or via its surface [38]. Although it has not yet been shown for GNE, which is particularly the case since no 3D structure is available for the entire enzyme, substrate channeling could also be a possibility for substrate handover here. 

**Figure 1:**
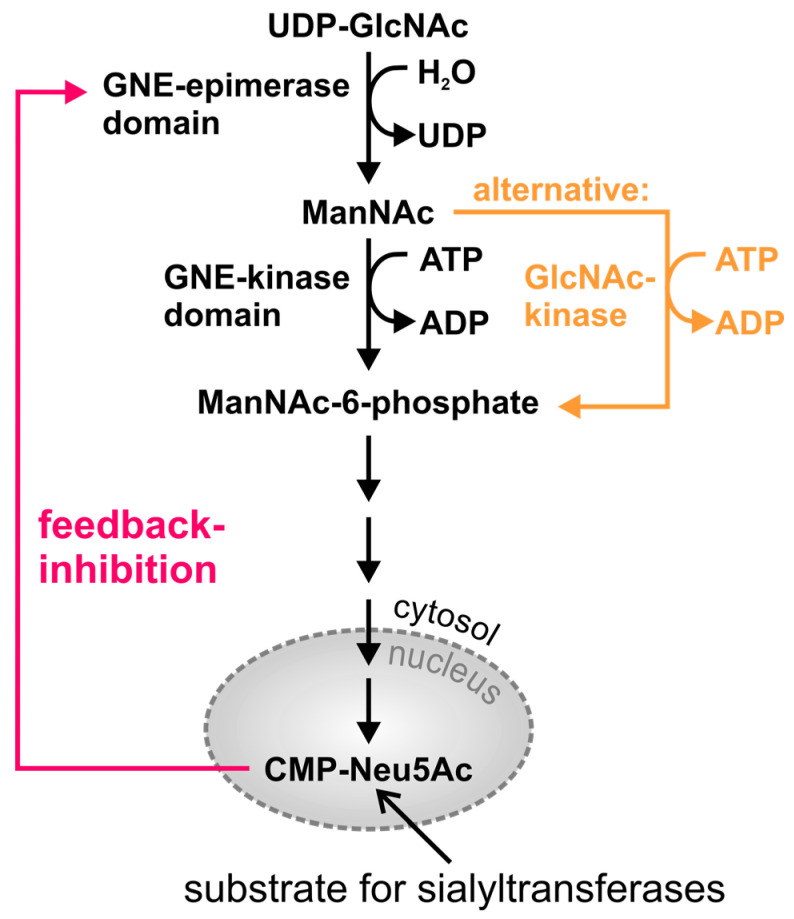
Schematic representation of the sialic acid biosynthetic pathway.

The epimerase domain catalyzes the formation of *N*-acetylmannosamine (ManNAc) from uridine diphosphate *N*-acetylglucosamine (UDP-GlcNAc) with a simultaneous cleavage of UDP [29,30] (Figure 1). The so-formed ManNAc is then phosphorylated by the kinase domain whereby ATP is converted to ADP at the same time [32]. Accordingly, the newly formed substance is called ManNAc-6-phosphate. Alternatively, this step can also be accomplished by the *N*-acetylglucosamine kinase (GlcNAc kinase/also known as NAGK; [40,41]; indicated in orange in Figure 1). The main substrate of this enzyme is usually GlcNAc, as supported, for example, by the corresponding *k_m_*-values for the two substrates ManNAc and GlcNAc [40,42]. 

After a few more steps (for a complete overview of all steps see [8,43]), cytidine 5′-monophospho-*N*-acetylneuraminic acid (CMP-Neu5Ac; [44,45]) is synthesized. CMP-Neu5Ac acts as substrate for sialyltransferases [46] and its bioavailability regulates their expression [47]. Additionally, it feedback-inhibits the activity of the epimerase ([48]; indicated by the red arrow in Figure 1). The allosteric site is located in the epimerase domain between the amino acids 255 and 303 (hGNE1) [14,43,49,50].

**Figure 2:**
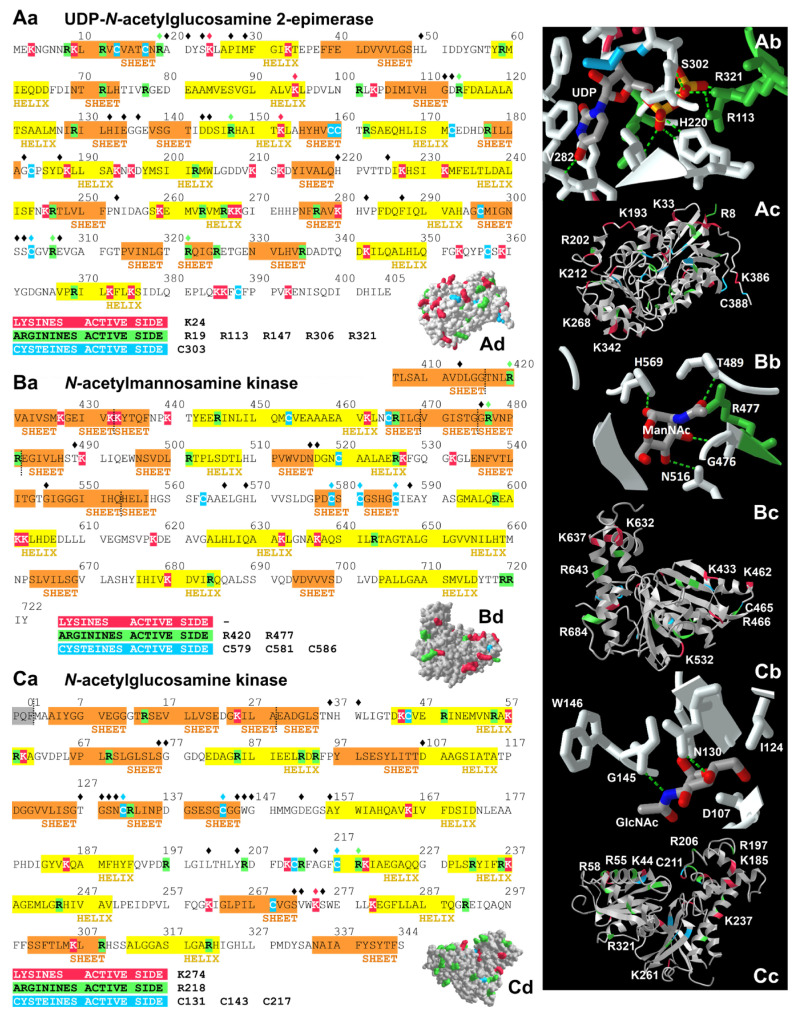
Localization of potential glycation sites in the proteins of interest: the two domains of the human GNE, mRNA variant 2 (hGNE1) and the *N*-acetylglucosamine kinase. Amino acid sequences of the GNE epimerase (**Aa**), GNE kinase domain (**Ba**), and of the *N*-acetylglucosamine kinase (**Ca**). Secondary structure elements are highlighted by an orange (sheet) or yellow (helix) background color. Lysines (red), arginines (green) and cysteines (blue) are also color-coded to indicate potential glycation sites. A diamond ♦ over an amino acid indicates whether it is within the active site. Lysines, arginines and cysteines found on the surface of the proteins are represented in **Ac**, **Ad**, **Bc**, **Bd**, **Cc**, and **Cd**. Interactions between the substrate and the active-site amino acids are indicated by green-dashed lines in **Ab**, **Bb** and **Cb**. All visualizations (PDB-IDs: 4ZHT (GNE epimerase domain; 3D structure published in [53]), 2YHW (GNE kinase domain; 3D structure published in [54]), 2CH5 (*N*-acetylglucosamine-kinase; 3D structure published in [56])) were created with iCn3D [79,80].

The amino acid side chains that are involved in substrate binding or in the formation and stabilization of the active site can be seen in Figure 2Aa,Ab (GNE-epimerase domain), Figure 2Ba,Bb (GNE-kinase domain), and Figure 2Ca,Cb (GlcNAc kinase) [sequences in 2Aa,Ba,Ca: amino acids involved are marked with a diamond ♦]. In the epimerase domain, these are R19, A20, D21, S23, K24, P27, M29, H49, G111, D112, R113, H132, E134, G136, D143, D144, R147, G182, D187, H220, D225, N253, V282, F287, S301, S302, C303, R306, E307 and R321 [43,50,51,52,53]. Please note: Since we show the amino acid sequence of hGNE1, the isoform examined in this study, in Figure 2, we also use the amino acid numbering based on this “old” variant. In the kinase domain, these are D413, R420, G476, R477, T489, N516, D517, G545, E566, H569, C579, C581, C586, and E588 [43,51,52,54]. In the GlcNAc-kinase, these are N36, W38, S76, G77, D107, T127, G128, S129, N130, C131, C143, G145, W146, G147, D152, A156, L201, Y205, A214, C217, R218, S271, V272, K274, and S275 [55,56]. 

GNEM is a distal myopathy with a worldwide predicted prevalence of 1 to 9 patients per 1 million people [8] that first affects the tibialis anterior, the biceps femoris short head, and the adductor muscles [57,58] leading to certain gait problems and a typical foot-drop [59,60]. After an average of 12 to 15 years after disease onset, most of the patients will be dependent on a wheelchair [61,62]. Other hallmarks of GNEM are the finding of rimmed vacuoles in affected muscles [60,63], an onset in early adulthood [62,64], and a quite slow progression of the disease [8,65]. GNEM-triggered inflammatory changes are not typical features of this disease [60,63,66], although there are confirmed exceptions to this statement [67,68,69].

Muscles/Muscle groups, which are least affected, are the quadriceps [60]—especially the vastus lateralis [57,58]—as well as the muscles in the face [8,59,63] and the deltoid muscle [60]. An influence on the heart and especially on cardiomyocytes is still under debate. While mouse models suggest an influence on cardiac muscle cells [70,71], patient data-based studies do not find direct correlations between GNEM and cardiomyopathy [61,64,72].

One therapeutic approach aims to compensate for potential hyposialylation by adding precursors of individual metabolites of the sialic acid pathway or other heavily sialylated proteins. This approach also led to positive effects in mouse models [8,70,73,74,75,76,77]. Unfortunately, the direct transferability of the results of the animal experiments did not turn out to be that easy [8,78]. Overall, although some trials are quite promising, the disease is not yet curable.

A particular sticking point of this disease, especially in the diagnosis, is the comparatively late onset of the disease. Other muscle disorders as Duchenne muscular dystrophy (DMD), Emery-Dreifuss muscular dystrophy (EDMD), or Facioscapulohumeral muscular dystrophy (FSHD) have an onset in early or late childhood [81]. This suggests that age-related effects may play a role in this disease. One hallmark of molecular aging is glycation. Glycation describes an enzyme-independent process of a post-translational modification (PTM) of proteins; in particular, of the amino acid side chains lysine, which has a primary amino group, arginine with its guanidine group [82], cysteine with its sulfhydryl group [83], or of other substances with a preferably terminal amino group [84].

To simplify the identification of potential glycation sites in the sequences of the GNE-epimerase domain, GNE-kinase domain, and GlcNAc kinase, we used a specific color code for arginines, cysteines, and lysines (Figure 2Aa,Ba,Ca). The same color code was also used in our three-dimensional representations of the enzymes—shown once in the ribbon (Figure 2Ac,Bc,Cc) and once in the molecular surface variant (Figure 2Ad,Bd,Cd). In particular, the amino acids that are part of the active site can strongly affect the activity of an enzyme—e.g., if they are mutated or otherwise modified, as they may be critical for stabilization of the binding site or for interaction with the substrate through hydrogen bonds. The functions of arginines, cysteines, and lysines that are part of the active site are listed in Table 1. 

Since it is already known that mutations at some of the positions of these amino acids lead to reduced enzyme activities [50,51,53,55], it seems very likely that glycations of these amino acids would also have an impact on enzyme activity. To complete this picture, it is important to mention that the activity of the GNE can be influenced by phosphorylation [85] and *O*-GlcNAcylation [86].

**Figure 3:**
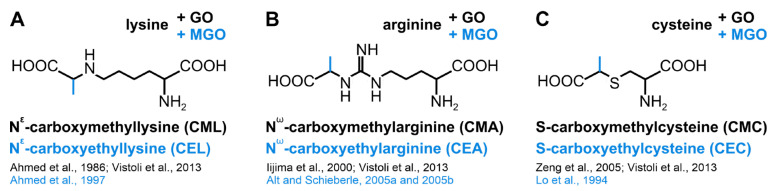
Structural formulas showing different advanced glycation end products (AGEs). Structural formulas of advanced glycation end products (AGEs) formed from lysine [95,108,109] (**A**), arginine [95,110,111,112] (**B**), or cysteine [83,95,113] (**C**) and glyoxal (GO; black) or methylglyoxal (MGO; blue).

The first step in glycation is a condensation between the amino group and a glycation agent, which can be, for example, a certain type of sugar [87], a derivate of sugar [88], di-carbonyls such as glyoxal (GO) or methylglyoxal (MGO) [89,90], or ascorbic acid [91]. A Schiff base adduct is formed, which can be converted into an Amadori product by an Amadori rearrangement [92]. This reaction can be followed by further steps—e.g., dehydration, enolizations, or oxidations [84,87,93]—leading to a multitude of possible advanced glycation endproducts (AGEs) [90,94]. The AGEs formed are also dependent on the glycation agent used ([95]; see also Figure 3A–C). Additionally, the reactivity of different amino acid side chains with different glycation agents varies [96]. 

The level of AGEs increases steadily over the course of life [97,98,99], which could possibly also have an influence on the symptomatic onset of GNEM. This hypothesis can be supported by the fact that there are already examples in which it has been shown that post-translational modifications caused by MGO lead to a decrease in the activity of an enzyme [100,101].

**Table 1:** Lysines, Arginines and Cysteines involved in the active site—Functions, Mutations, and Activity.

**Residues**	**Function**	**So-Far Known Mutations in Patients**	**In Vitro—Loss of Activity Due to Mutations (Compared to WT)**
UDP-*N*-acetylglucosamine 2-epimerase (hGNE1, mRNA variant 2)			
K24	Involved in forming the vicinity of the active site [52]	n.d.a.	n.d.a.
R19	UDP binding [52]	n.d.a.	n.d.a.
R113	Involved in forming the vicinity of the active site [52]	n.d.a.	R113A: completely inactive [53]
R147	Involved in forming the vicinity of the active site [52]	n.d.a.	n.d.a.
R306	Involved in forming the vicinity of the active site [50]	R306Q [102]	n.d.a.
R321	Interaction with the phosphate of UDP [53]	R321C [103]	n.d.a.
C303	Hydrophobic interactions, but probably no specific function in the enzymatic reaction [50]	C303V [104] C303X [9]	C303V: epimerase 80%, kinase 60% C303X: epimerase 0%, kinase 0% [50]
*N*-acetylmannosamine kinase (hGNE1, mRNA variant 2)			
**Residues**	**Function**	**So-Far Known Mutations in Patients**	**In Vitro—Loss of Activity Due to Mutations (Compared to WT)**
R420	Interaction with the phosphate oxygens [51,52]	R420X [105] R420Q [106] → patient not yet suffering from myopathy	R420M: epimerase activity comparable to WT; kinase activity drastically reduced [51]
R477	ManNAc binding [54]	n.d.a.	n.d.a.
C579	Zinc binding [54]	C579Y [12]	n.d.a.
C581	Zinc binding [54]	C581R [7]	n.d.a.
C586	Zinc binding [54]	C586X [7]	n.d.a.
*N*-acetylglucosamine kinase			
**Residues**	**Function**	**In Vitro—Loss of Activity Due to Mutations (Compared to WT)**	
K274	Nucleotide binding [56]	n.d.a.	
R218	Nucleotide binding [56]	n.d.a.	
C131	Phosphate transfer from ATP to the hydroxyl groups of GlcNAc [55]	C131S: overall activity reduced (approx.. 10% activity remaining [55])	
C143	Phosphate transfer from ATP to the hydroxyl groups of GlcNAc [55]	C143S: overall activity reduced (approx.. 20% activity remaining [55]); 75% of WT activity [107]	
C217	Nucleotide binding [56]	C217S: overall activity reduced (approx.. 30% activity remaining [55])	

n.d.a.: no data available.

The aforementioned di-carbonyl substances can occur naturally as a breakdown product of glycolysis, amino acid, and fatty acid degradation [114,115,116]. In addition, they can arise as a byproduct of the glycation process itself via the so-called Namiki pathway [84,117] or by autoxidation of glucose via the Wolff pathway [84,118,119,120]. They are also ingested through food: MGO and GO, e.g., by consuming coffee and wine [121] and Vitamin C through fruits and vegetables [122]. 

One of the most well-studied AGEs is carboxymethyllysine (CML; Figure 3A), whose formation is based primarily on a reaction between lysine and glyoxal [108,123,124]. Furthermore, most anti-AGE antibodies preferentially recognize CML [125]. In our study, we also used an antibody against CML as a marker for the glycation level of our proteins—the epimerase and the kinase domain of the GNE and the GlcNAc kinase. 

In this study, we attempted to find an explanation for the late onset of GNEM by relating it to PTMs, focusing specifically on glycation. Therefore, we investigated the extent of the influence of MGO- or GO-triggered glycation on the activity of the individual domains of the GNE (hGNE1) and on the GlcNAc kinase. For this purpose, all three enzymes were expressed separately from each other, purified, treated with increasing concentrations of one of the glycation agents, and then analyzed for their activity. 

Furthermore, we examined the expression of the GNE—as a whole protein—and the GlcNAc kinase in undifferentiated but MGO- or GO-treated C2C12 cells. These cells belong to a murine skeletal muscle cell line. In addition, the effect of the glycation agents on cell viability was investigated by a MTT assay. 

## 2. Materials and Methods

### 2.1. Cell Culture and MGO/GO-Treatment

Murine C2C12 myoblasts were cultured in DMEM (Dulbecco’s Modified Eagle’s Medium; 11960044; Gibco/Thermo Fisher Scientific; Waltham, MA, USA) supplemented with 10% FBS (Fetal Bovine Serum; A5256801; Gibco/Thermo Fisher Scientific; Waltham, MA, USA), 1% penicillin-streptomycin (P/S; 10,000 units/mL (P) and 10,000 µg/mL (S); 15140122; Gibco/Thermo Fisher Scientific; Waltham, MA, USA) and 1% L-glutamine (L-Gln; 200 mM; A2916801; Gibco/Thermo Fisher Scientific; Waltham, MA, USA) at 37 °C in a humidified atmosphere with 5% CO_2_. The C2C12 cell line was kindly provided to us by the Poserns Lab (Martin-Luther-University Halle-Wittenberg, Institute for Physiological Chemistry; Halle (Saale), Germany). Growth medium was changed every 48 h. 

To assess the effect of MGO and GO on C2C12 myoblasts, the cells were seeded at a density of 9000 cells/cm^2^; in a 10 cm (Ø) dish and incubated at 37 °C in a humidified atmosphere with 5% CO_2_. After one day, the cells were cultured in starvation medium (DMEM + 1% FBS + 1% P/S + 1% L-Gln) with MGO or GO (0.2 mM, 0.5 mM, or 2 mM). Following another day, the cells were divided into two pellets, one for protein isolation and one for RNA isolation.

### 2.2. RNA Extraction and RT-qPCR

Total RNA was isolated using the Quick-RNA Miniprep Kit (R1054; Zymo Research; Irvine, CA, USA). cDNA was synthesized, using 2 µg of total RNA and SuperScript™ II reverse transcriptase (18064022; 2000 units; Thermo Fisher Scientific; Waltham, MA, USA), following the manufacturer’s instructions. RT-qPCR was performed using qPCR SybrMaster (PCR-372S; Jena Bioscience; Jena, Germany) and the CFX Connect™ Real-Time PCR Detection System (1855201; Bio-Rad; Hercules, CA, USA). 

The following primers were used: 


**Gene Name**

**Direction**

**Sequence**
RPL26forwardGGTCTATGCCCATTCGGAAGGRPL26reverseTCGTTCGATGTAGATGACGTACTGAPDHforwardCCTGGAGAAACCTGCCAAGTATGGAPDHreverseAGAGTGGGAGTTGCTGTTGAAGTCIsoform 1 (short)forwardGGCGTCCGGGTTCTACGCAIsoform 2 (long)forwardGGAAACACACGCGCATCTCCACIsoform 1 and 2reverseAATGGTCCCGCTGACCTCGCGNE1forwardGGTGGACAATGACGGCAACTGTGNE1reverseCAGTTCGTGCTGGTGGATGATCGlcNAc kinaseforwardGGTAGTATGGCCGCGCTTTAGlcNAc kinasereverseGGTGTGCAATCCAGTAGGCT

Cq values were normalized to the housekeeping gene RPL26, and relative gene expression was calculated using the ΔΔCq-method.

### 2.3. Protein Isolation from Cell Culture 

For protein isolation, cells were washed with PBS and lysed using RIPA buffer containing protease inhibitor cocktail (Sigma Aldrich; St. Louis, MO, USA), 1 mM NaVO_4_, and 1 mM PMSF. Following 30 min incubation on ice, total protein was isolated by centrifugation at 14,000× *g*, 4 °C for 5 min and quantified using the Pierce™ BCA Protein Assay Kit (23225; Thermo Fisher Scientific; Waltham, MA, USA).

### 2.4. MTT Assay

C2C12 myoblasts were seeded at a density of 3 × 10^3^ cells in a 96-well cell culture plate and treated with six different concentrations of MGO (0 mM, 0.1 mM, 0.2 mM, 0.5 mM, 0.7 mM, and 1 mM) and GO (0 mM, 0.5 mM, 1 mM, 1.5 mM, 2 mM, and 2.5 mM) for 24 h in DMEM containing 1% FBS. Thiazolylblue-tetrazolimbromide (M5655; Sigma-Aldrich; St. Louis, MO, USA) was added to the cells according to the manufacturer’s instructions and incubated for 4 h.

### 2.5. Protein Expression in E.coli and Purification

The GNE domains of human GNE/MNK were expressed according to a protocol published by the Chen Group [53]. The gene encoding the UDP-GlcNAc 2-epimerase domain with an N-terminal His6-tag was bought from Biocat in a pET21a(+) vector. The plasmid was codon optimized to be expressed in Escherichia coli BL21 (DE3) cells. The transformed BL21 (DE3) cells were stored until use as glycerol stocks (5%). 

Starting from this glycerol stock, an over-day culture was grown in 5 mL LB medium with 100 mg/L ampicillin, for 8 h at 37 °C and 220 rpm. From the over-day culture, an overnight culture was grown in 15 ml LB medium with 100 mg/L ampicillin, at 37 °C and 220 rpm. With 3.5 mL overnight cultures 4 × 500 mL LB medium with 100 mg/L ampicillin in 2 L flasks were inoculated. This was grown at 37 °C and 180 rpm to an OD600 of 1.9. Then all cultures were pooled, and 700 mL were diluted in 1.3 l LB medium with 100 mg/L ampicillin (at 4 °C). This was redistributed to four 2 L flasks (4 × 500 mL), grown to an OD600 of 0.7 and induced with IPTG (50 µM). Subsequently, the cells were kept for 42 h at 8 °C and 120 rpm. Then, they were grown for 24 h at 16 °C and 180 rpm. The cells were harvested by centrifugation with 4000× *g* for 15 min at 4 °C, and the cell pellet was re-suspended in resuspension buffer (50 mM Tris-HCl, pH 8.0, 500 mM NaCl).

Afterwards, the cells were lysed by using a high shear fluid laboratory homogenizer at 18 kPa (LM10; Microfluidics International Corporation; Newton, MA, USA) and the cell lysate was centrifuged with 20,000× *g* for 25 min at 4 °C. The clarified supernatant was filtered, and the protein was purified via Ni-NTA chromatography on 2 × 5 mL His60 Ni Superflow column (635657; Takara Bio Inc./Clontech; Kusatsu, Japan) using a Chromatography System (Bio-Rad NGC Discover 10; Bio-Rad Laboratories; Hercules, CA, USA). Resuspension buffer and elution buffer (50 mM Tris-HCl pH 8.0, 500 mM NaCl, 500 mM Imidazole) were used in a linear gradient over 300 mL. 

Fractions of the protein peak were pooled and concentrated to 10 mL using a Vivaspin 20 (VS2001; Sartorius; Göttingen, Germany) with a molecular weight cut of 10 kDa. The sample was then buffer exchanged to 50 mM Tris-HCl pH 8.0, 100 mM NaCl, 5% glycerol and 0.2 mM TCEP using a HiPrep 26/10 Desalting column (GE17-5087-01; Sigma-Aldrich; St. Louis, MO, USA) and the aforementioned chromatography system. Fractions containing the GNE protein were pooled. 20 mL with a concentration of about 7 mg/mL were obtained.

To avoid confusion with the proteins from cell culture, these proteins will be referred to hereinafter as Protein-Ecoli and the proteins from cell culture as Protein-CC.

### 2.6. Glycation of the Proteins (Protein-Ecoli)

In order to investigate the concentration dependence of glycation of methylglyoxal (MGO; 40% in H_2_O; Sigma Aldrich; St. Louis, MO, USA) and glyoxal (GO; 40% in H_2_O; Sigma Aldrich; St. Louis, MO, USA), 6 µg each of *N*-acetylmannosamine kinase and *N*-acetyl-glucosamine kinase or 4.8 µg of the UDP-*N*-acetylglucosamine 2 epimerase were mixed with ascending concentrations of the reagents—0.5 mM, 2 mM, and 5 mM—and incubated for 1 h at 37 °C. 

### 2.7. Western Blot Analysis (Protein-CC and Protein-Ecoli)

Protein-CC:

Equal amounts of protein were mixed with 5× SDS-loading dye (containing 50 mM DTT) and separated on a 4–12% Tris-Glycin gradient gel (Invitrogen by Thermo Fisher; Waltham, MA, USA). 

Protein-Ecoli:

The glycated proteins were mixed with 5× SDS-loading dye (containing 50 mM DTT) and separated on a 10–20% Tris-Glycin gradient gel (XP10205BOX; Invitrogen by ThermoFisher; Waltham, MA, USA). 

Proteins were transferred on a nitrocellulose-membrane and stained with Ponceau S as loading control. Membranes were blocked with 5% skimmed milk in TBS-Tween (TBS-T) for 1 h at RT. The membranes were then incubated with the primary antibodies: mouse IgG-anti-carboxymethyl lysine (CML26; dilution: 1:10,000 (Protein-CC) or 1:2000 (Protein-Ecoli); ab125145; abcam; Cambridge, UK) or MG-H1 (1H7G5; dilution: 1:1000; NBP2-62810; Novus Biologicals/Bio-Techne; Minneapolis, MN, USA) overnight at 4 °C. Afterwards, the membranes were washed three times with TBS-T. Each washing step lasts 10 min. Following that, the membranes were incubated with the secondary antibody—goat anti-mouse IgG H&L HRP (dilution 1:10,000; ab6789; abcam; Cambridge, UK)—for 1 h at RT. Thereafter, the membranes were again washed 3 times for 10 min each with TBS-T and the Western blot detection reagent from Amersham (cytiva, Marlborough, MA, USA) was used. The ChemiDoc MP imaging system from Bio-Rad Laboratories (Hercules, CA, USA) was used for visualization. Intensities were quantified by ImageLab Software from Bio-Rad Laboratories (Hercules, CA, USA).

### 2.8. Epimerase Activity Assay (Protein-Ecoli)

An UDP Glo™ Glycosyltransferase Assay (V6961; Promega Corporation; Madison, WI, USA) was performed to determine UDP-*N*-acetyl-glucosamine 2-epimerase activity. For this purpose, 747 µg epimerase were incubated at 37 °C with different MGO/GO concentrations (0.5 mM, 2 mM, and 5 mM) for 1 h in a volume of 3 µL. The total volume of the reaction mixture of 52.5 µL was prepared, consisting of 5 µM UDP-GlcNAc, 0.05% BSA, and glycated epimerase in buffer. This mix was incubated for 1 h at 37 °C. Afterwards, 25 µL UDP-Detection Reagent™ were added and incubated at room temperature for 1 h. Afterwards, the luminescence was detected by ClarioStar™ (BMG Labtech GmbH, Ortenberg, Germany).

### 2.9. Kinase Activity Assay (Protein-Ecoli)

The *N*-acetylmannosamine kinase activity and the *N*-acetylglucosamine kinase activity were determined by a coupled enzyme assay (based on the assays described by [19,126]). 

Our enzyme assay used differently glycated GNE-kinase and GlcNAc kinase samples. For this, 3 µg each of the GNE-kinase domain and the GlcNAc kinase were incubated with ascending concentrations of MGO and GO—0.5 mM, 2 mM, and 5 mM—for 1 h at 37 °C. Each of the enzyme samples had a total volume of 2.5 µL. 

Afterwards, a reaction mixture with a total volume of 623.5 µL was prepared, consisting of one of each of the different enzyme samples, 62.6 mM Tris, 20.3 mM MgCl_2_, 9.6 mM ATP, 4.8 mM ManNAc, 4.8 mM phosphoenolpyruvate, 1.4 mM NADH, 6 µL Lactic dehydrogenase (600–1000 U/mL)/Pyruvat kinase solution (900–1400 U/mL) (P0294; Sigma Aldrich; St. Louis, MO, USA), and 7.7 mM sodiumphosphatebuffer. 

This reaction mixture was incubated at 37 °C until further use. At time points 0 min and 45 min in the case of the ManNAc kinase, and 0 min and 180 min in the case of the GlcNAc kinase, 25 µL of this mixture was removed and added to 75 µL of an EDTA solution (10 mM). The absorbance at the wavelength of 340 nm reflects the NADH concentration. The ClarioStar™ (BMG Labtech GmbH, Ortenberg, Germany) was used to determine the absorbance.

### 2.10. Structural Comparison

A structural comparison between GNE-kinase and GlcNAc kinase was performed using the VAST+ algorithm (vector alignment search tool; [127,128,129]).

In order to do this, we entered the PDB ID of the GNE-kinase (2YHW) into the search field of the Website of the National Center of Biotechnology Information [130], called up the entry listed under Protein/Structure, and then clicked on the “VAST+” button (similar structures). Afterwards, we entered the PDB ID of the GlcNAc kinase (2CH5) into the search field of the result list.

### 2.11. Statistical Analysis

For statistical analysis of the epimerase and the kinase activity assays, a two-way analysis of variance (ANOVA) was performed (alpha = 0.05; OriginPro 2019 (OriginLab Corporation; Northampton, MA, USA)). One factor (“way”) was the choice of glycation agent—MGO or GO—and the other the concentration used—0.5 mM, 2 mM, or 5 mM. This was followed by a Tukey post hoc-Test. Based on the determined *p*-values, asterisks were used to classify the significance levels: *p* ≤ 0.05: *, 0.005 < *p* ≤ 0.01: **, and *p* ≤ 0.005: ***.

## 3. Results

### 3.1. Expression and Glycation of the UDP-N-Acetylglucosamine 2-Epimerase, the N-Acetylmannosamine kinase, and the N-Acetylglucosamine Kinase

As it is possible that molecular aging is an important component in the progression and onset of GNEM, this aspect will be the focus of the following experiments. Molecular aging was therefore simulated by glycating the proteins/protein domains of interest using different physiological glycation agents. Afterwards, their individual activities were determined by different assays further explained in the next result section. 

**Figure 4:**
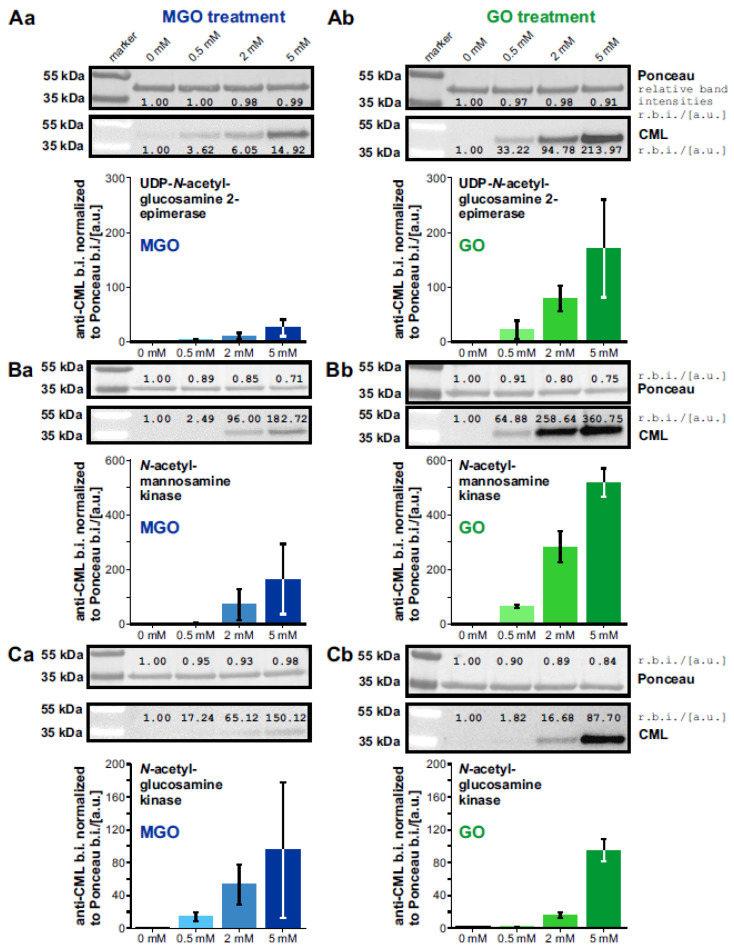
Investigation of the glycation success of the two GNE-domains and the GlcNAc kinase. The glycation success on each protein/protein domain was investigated through western blots by using an anti-CML antibody specifically against glycated lysines—or more precisely against carboxy-methyl-lysines. (**A**) shows the glycation of the UPD-*N*-acetylglucosamine 2-epimerase by methylglyoxal (MGO; **Aa**) or glyoxal (GO; **Ab**). Correspondingly, (**B**) shows the glycation of *N*-acetylmannosamine kinase by MGO (**Ba**) or GO (**Bb**) and (**C**) that of *N*-acetylglucosamine kinase by MGO (**Ca**) or GO (**Cb**). For each protein/protein domain, a representative Ponceau stain, a representative Western blot, and a bar graph with the anti-CML band intensities normalized to Ponceau (n = 2) were shown. At a concentration of 0 mM, buffer was added instead of a glycation agent. The relative band intensities were written directly above or underneath each band.

First, the proteins/protein domains were expressed in *Escherichia coli* BL21 with a 6xHis-tag added to their N-terminal site. This His-tag was used for purification over Ni-NTA-columns (details can be found in the Material and Methods section). Afterwards, the purified proteins were incubated with ascending concentrations of the glycation agents methylglyoxal (MGO) and glyoxal (GO) and the glycation success was verified by western blots using an antibody specific for glycated lysine side chains (anti-CML; see Figure 4, Supplementary Table S1 and the Supplementary Figures S1–S3 (showing the uncut Western Blots)). 

With ascending concentrations of MGO and GO, the level of relative glycation normalized to Ponceau increases. Across both GNE domains, the signal of CMLs detected was stronger when using the glycation agent GO than MGO. 

Looking at the GlcNAc kinase, it is striking that the amount of CMLs and thus the glycation success seems to be at the same level for both glycation agents. Overall, the proteins/protein domains of interest were successfully glycated by both glycation agents.

### 3.2. Glycation of the GNE-Domains Interferes with Their Enzymatic Activity—Activity of the GlcNAc Kinase Is Not Affected by Glycation

Having shown that the proteins/protein domains of interest were successfully glycated, their activity should now be determined. The aim is to investigate whether glycation can influence enzyme activity, to be able to assess whether the late onset of GNEM might be due to age-related effects.

**Figure 5:**
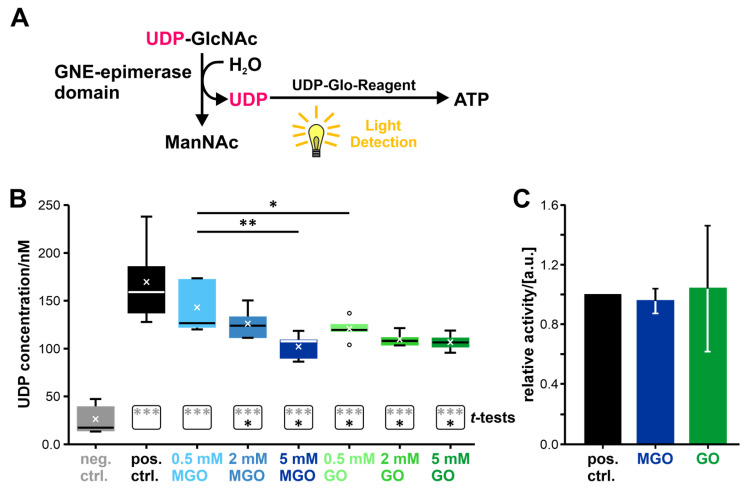
Investigation of the influence of glycation on the activity of the GNE-epimerase domain by a luminescence-based assay. (**A**) Scheme of the enzyme activity test with all the relevant reactions. (**B**) Boxplot showing the determined UDP concentration (n = 5; to minimize technical noise, three technical replicates were made for each biological replicate and these values were then averaged → N = 3). (**C**) Bar graph showing whether the glycation agent affects the functionality of the assay (n = 3; N = 3). No GNE-epimerase domain was added in this part, only the assay substances, UDP and the glycation agent (MGO or GO). The abbreviation “a.u.” stands for arbitrary unit. The median was included in all quantile calculations and is represented by a line in the boxplots. The mean is indicated by x’s. Outliers are marked with circles. The boxes below the boxplots show the student’s *t*-test results for each condition. Grey asterisks indicate the results versus the negative control and black asterisks versus the positive control. *p* ≤ 0.05: *, 0.005 < *p* ≤ 0.01: **, and *p* ≤ 0.005: ***.

The first step of the GNE-epimerase activity assay is based on the GNE-epimerase domain reaction, where UDP-*N*-acetylglucosamine (UDP-GlcNAc) is converted under addition of water to *N*-acetylmannosamine (ManNAc) and UDP (see Figure 5A). Afterwards, the generated UDP is converted to ATP by adding UDP-Glo-Reagent and light is generated in the form of luminescence (based on the Promega glycosyltransferase assay; details can be found in the Material and Methods section). The amount of light produced was used to back-calculate the UDP concentration. This is possible because the UDP concentration and the amount of light produced are proportionally coupled. The determined UDP concentration was then used as an indicator for enzyme activity. Higher UDP concentrations indicate higher enzyme activities. 

The boxplot showing the determined UDP concentrations under the different conditions can be seen in Figure 5B. All boxplot-related data, including mean and standard deviation, can be found in Supplementary Table S2. All samples showed significant differences (*p* < 0.005; student’s *t*-test; all results can be seen in Supplementary Table S5) towards the negative control (see Figure 5B). The negative control consists of water, instead of any enzyme. The positive control consists of the non-glycated enzyme. With the exception of the sample that was treated with 0.5 mM MGO, all other samples showed significant differences towards the positive control (*p* < 0.05; student’s *t*-test), indicating reduced enzyme activities—lower UDP-concentrations—compared to the non-glycated enzyme. 

Two-factor ANOVA showed that the concentration of the glycation agent is a significant factor for the activity (*p*-value: 1.43E-03; see also Supplementary Table S3) and the choice of the glycation agent *per se* not (*p*-value: 1.96E-01). The interaction between concentration and glycation agent is again significant (*p*-value: 3.58E-03), meaning that the result obtained through different concentrations depends on the used glycation agent. 

The subsequent Tukey post hoc-Test identified significant differences between the conditions 0.5 mM MGO and 2 mM MGO, between the conditions 0.5 mM MGO and 0.5 mM GO, and between the concentrations 0.5 mM and 5 mM, regardless of the glycation substance used (for the *p*-values see Supplementary Table S4).

To ensure that the glycation agents do not influence the activity assay itself, the experiment was repeated without the GNE-epimerase domain, but with 0.25 nmol UDP (see Figure 5C). The assay enzymes were exposed to one of the two glycation agents in the same amount as in the normal activity assay (same volume; always assuming the highest concentration used). No influences on the activity of the assay enzymes were found under these conditions (*p*-value: 5.35E-01 (control and MGO); *p*-value: 9.07E-01 (control and GO)). This indicates that the reduced UDP concentrations measured with differentially glycated GNE-epimerase domains were a result of a reduced GNE-epimerase domain enzyme activity and not a result of the glycation agents reacting with the assay itself.

In summary, it can be stated that the activity of the GNE-epimerase domain can be influenced by glycation, with a remaining activity of 60% ± 8% after treatment with MGO (5 mM) and 63% ± 5% after treatment with GO (5 mM) compared to the non-glycated protein, which activity was set to 100%. The determination of the activity is based on the UDP consumption (see Supplementary Table S2). This also shows that regardless of the glycation agent used, the same level of activity is achieved when comparing the highest glycation conditions. 

**Figure 6:**
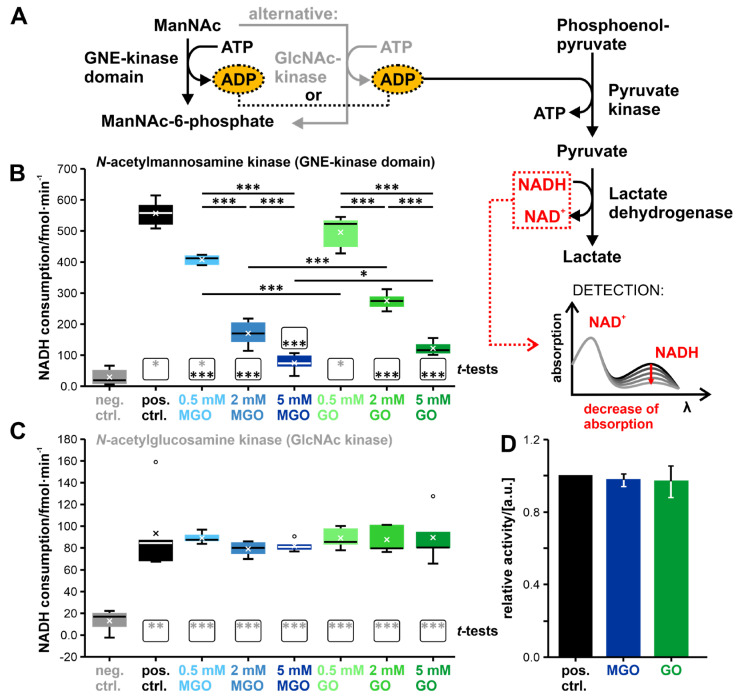
Investigation of the influence of glycation on the activity of the GNE-kinase domain and of the GlcNAc kinase by a coupled enzyme assay. (**A**) Scheme of the enzyme activity test with all the relevant reactions. (**B**) Boxplot showing the determined NADH consumption per minute of the GNE-kinase domain (n = 5). (**C**) Boxplot showing the determined NADH consumption per minute of the GlcNAc kinase domain (n = 5). (**D**) Bar graph showing whether the glycation agent affects the functionality of the assay (n = 3). The abbreviation “a.u.” stands for arbitrary unit. The median was included in all quantile calculations and is represented by a line in the boxplots. The mean is indicated by x’s. Outliers are marked with circles. The boxes below the boxplots show the student’s *t*-test results for each condition. Grey asterisks indicate the results versus the negative control and black asterisks versus the positive control. *p* ≤ 0.05: *, 0.005 < *p* ≤ 0.01: **, and *p* ≤ 0.005: ***.

The activities of the GNE-kinase domain and of the GlcNAc kinase were determined by a coupled enzyme assay ([19,126]; see Figure 6A). This assay is based on the fact that both kinases can phosphorylate ManNAc to ManNAc-6-phosphate, although ManNAc is not the main substrate of GlcNAc kinase [40,42]. The ADP, which remains after the release of the phosphate from the ATP, is then further used in the pyruvate kinase reaction. The product of that reaction, pyruvate, is further converted to lactate. Concomitantly, the coenzyme NADH is converted to NAD^+^. The decrease in the NADH concentration can be measured by determining the absorbance at 340 nm. The level of NADH consumption per minute can be seen as a measure of enzyme activity. Higher NADH consumption per minute indicates higher enzyme activities. 

The determined NADH consumption per minute for the GNE-kinase domain can be seen in Figure 6B and for the GlcNAc kinase in Figure 6C (the boxplot-related data can be found in the Supplementary Tables S6 and S10). The GlcNAc kinase, even non-glycated, shows a lower NADH consumption rate per minute than the GNE-kinase domain—only 17% compared to the GNE-kinase domain. 

Concerning the GNE-kinase domain, the positive control—again consisting of non-glycated protein—and the samples treated with the lowest concentrations of the glycation agents showed significant differences towards the negative control (*p* < 0.05; student’s *t*-test; see Figure 6B and Supplementary Table S8). All other samples showed such low NADH consumptions that they no longer show any significant differences from the negative control. With the exception of the treatment with 0.5 mM GO, all samples showed a significant difference from the non-glycated sample (pos. control; *p* < 0.005; see also Supplementary Table S8). The overall trend related to the NADH consumption per min is that higher concentrations of the glycation agent leads to lower consumption rates.

The two-factor ANOVA showed that the concentration of the glycation agent and the glycation agent per se are significant factors (*p* < 0.005; see also Supplementary Table S7) and the interaction between them is not (*p*-value: 1.22 × 10^−1^). 

The subsequent Tukey post hoc-Test identified many significant differences between the different conditions, which can all be seen in Figure 6B with the corresponding *p*-values in Supplementary Table S9. 

Concerning the GlcNAc kinase, all samples differ significantly from the negative control (*p* < 0.01; see Figure 6C and Supplementary Table S13) and show no significant difference from the positive control. Additionally, all other statistical tests do not reveal any other significant effects (see Supplementary Tables S10 and S11). This is quite surprising, as it states that glycation does not affect the enzymatic activity of this kinase, despite previous results on the GNE-kinase domain suggesting otherwise.

Again, no influence of the glycation agents on the enzymes of the assay could be determined (see Figure 6D; *p*-value: 4.23 × 10^−1^ (control and MGO); *p*-value: 6.52 × 10^−1^ (control and GO)). We exposed the assay enzymes to the same amounts of the two glycation agents as in the normal activity assay (same volume; always assuming the highest concentration used). 

### 3.3. Expression of the UDP-N-Acetylglucosamine 2-Epimerase/N-Acetylmannosamine Kinase, and the N-Acetylglucosamine Kinase in MGO/GO-Treated Undifferentiated C2C12 Cells

We also investigated the effect of MGO and GO on undifferentiated C2C12-cells, a murine skeletal muscle cell line. This should help to obtain a first impression of the effect of glycation on the muscle type that is most affected by the disease. Phase-contrast images of C2C12 cells treated with different concentrations of the glycation agents were then analyzed to find out which concentrations appear to be quite well tolerated. An MTT assay was then performed to determine the effect of the glycation agents on cell viability. Based on the data from the MTT assay, the TC_50_-value was calculated whenever possible. Finally, the effect of MGO and GO on mRNA expression of GNE and GlcNAc kinase was determined using quantitative PCR (qPCR).

**Figure 7:**
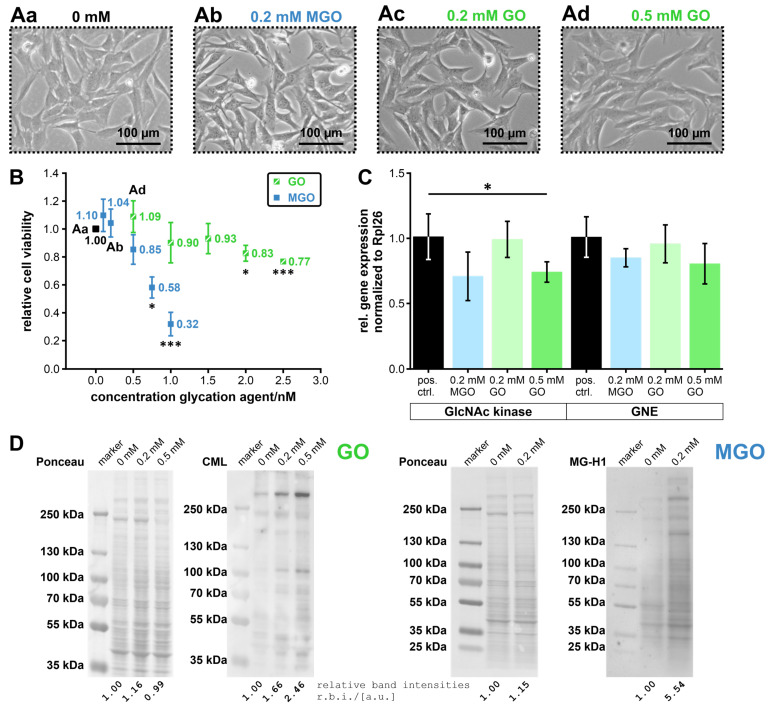
Investigation of the effect of the two glycation agents, MGO and GO, on C2C12-cells with a special focus on the expression of the GNE and the GlcNAc kinase. (**A**) Representative phase contrast images of C2C12 cells, which were treated with different concentrations of the two glycation agents (partly improved in contrast and brightness (the unprocessed images can be found in supplementary Figure S4)). The cells in (**Aa**) were not treated with either MGO or GO, the cells in (**Ab**) were treated with 0.2 mM MGO, the cells in (**Ac**) with 0.2 mM GO, and the cells in (**Ad**) with 0.5 mM GO. Furthermore, (**B**) shows the influences of MGO (blue) and GO (green) on the cell viability of the C2C12 cells, determined by an MTT assay (n = 3), and (**C**) on the mRNA expression of the GNE and the GlcNAc kinase determined by qPCR (n = 4). (**D**) Representative Ponceau stains and western blots showing GO- and MGO-derived glycation products (n = 4). GO-derived glycation products were detected by using an anti-CML antibody and MGO-derived glycation products by using an MG-H1-antibody (immunogen: N^α^-acetyl-N^δ^-(5-hydro-5-methyl)-4-imidazolone). The band intensities were determined as the sum of all intensities per lane. Relative band intensities were written directly under each column. *p* ≤ 0.05: * and *p* ≤ 0.005: ***.

Looking at the phase-contrast images revealed that the cells tolerated higher concentrations of GO than of MGO. Accordingly, we found no morphological abnormalities in the cell cultures at a maximum concentration of 0.2 mM MGO and 0.5 mM GO (see Figure 7A and Supplementary Figure S4 (showing the unprocessed cell culture images)). This can be confirmed by the results of the MTT assay (see Figure 7B; results and *p*-values can be found in Supplementary Tables S14 and S15), where we determined a TC_50_-value for MGO of 0.81 mM. With regard to GO, we decided against calculating this value based on the underlying data basis, since we did not exceed the 50% cell viability mark with the concentrations examined. Nevertheless, it is clear that the TC_50_-value of GO with respect to C2C12 myoblasts must be higher than the TC_50_-value of MGO. 

The western blots showed the same trend as previously observed when examining the proteins/protein domains individually—that with ascending concentrations of MGO and GO, the level of relative glycation increases (see Figure 7D). This also stays true after normalization to Ponceau (see supplementary Figure S5).

The qPCR showed that the mRNA expression of the GlcNAc kinase was significantly reduced after treatment with 0.5 mM GO (*p*-value: 4.56 × 10^−2^; see Figure 7C and Supplementary Table S16). The expression of the GNE showed no significant changes independent of the concentration or type of the glycation agent (all *p*-values can be found in Supplementary Table S17). 

Overall, these investigations showed that too high concentrations of glycation agents, especially of MGO, lead to a reduced cell viability. At non-toxic concentrations, GO/MGO-derived glycation products could be successfully detected in the cells/cell lysates; increasing concentrations led to increasing amounts of glycation products. The mRNA expression of GlcNAc kinase in C2C12 cells was affected by the addition of GO. However, the mRNA expression of GNE was not affected by either of the two glycation agents investigated.

### 3.4. Structural Comparison of the UDP-N-Acetylglucosamine 2-Epimerase/N-Acetyl-mannosamine kinase and the N-Acetylglucosamine Kinase 

**Figure 8:**
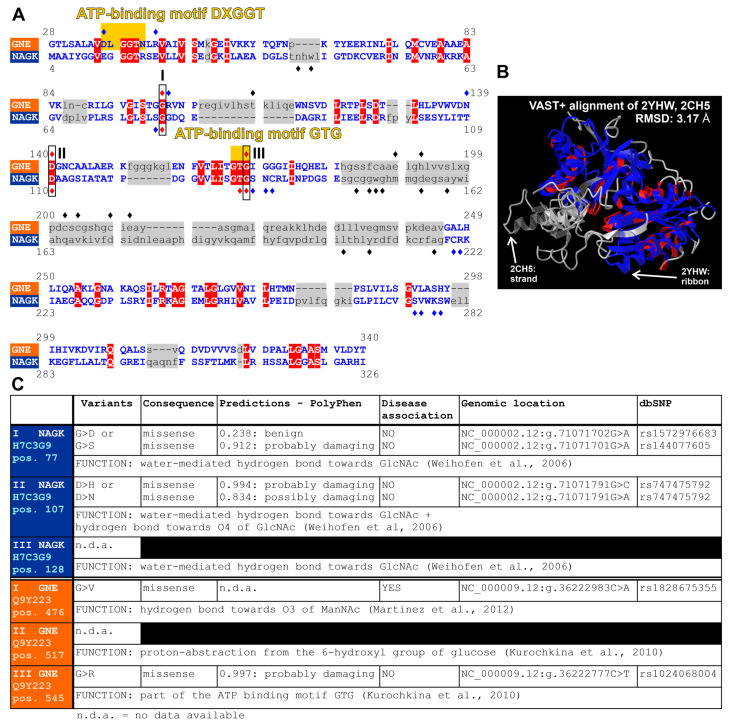
Structural comparison of the GNE-kinase domain (hGNE1) and the GlcNAc-kinase. (**A**) Amino acid comparison of GNE-kinase domain (orange) and GlcNAc kinase (blue). (**B**) Three-dimensional structural comparison of the two kinases. Both were based on the vector alignment search tool VAST+ [127,128,129]. A blue color represent amino acids that are aligned in 3D space, a gray color represent unaligned amino acids, and a red color represent identical amino acids. A diamond ♦ over an amino acid indicates whether it is within the active site. The three amino acids, which can be aligned, which are identical, and which are part of the active site are marked by Roman numerals from I to III. The amino acid marked with III—glycine—is part of the ATP-binding motifs DXGGT and GTG [52,131]. All other functions of the marked amino acids can be found in the table in (**C**). Most of the information given here was based on data found on https://www.uniprot.org, (accessed on 20 December 2022) [133].

To understand similarities and differences between GNE-kinase and GlcNAc kinase, a structural comparison of both was performed using the VAST+ algorithm (vector alignment search tool; [127,128,129]; see Figure 8). The root mean square deviation (RMSD) of the aligned residues between both kinases was 3.17 Å. Furthermore, a sequence accordance of 21% was found (displayed by white capital letters with a red background in Figure 8A; or by a red color in the three-dimensional image alignment in Figure 8B), and 219 residues could be aligned in 3D space (displayed by blue capital letters in Figure 8A; or by a blue color in Figure 8B). Regions in a gray color (Figure 8B) or with lowercase letters with a gray background represent unaligned amino acids (Figure 8A). In addition, a diamond ♦ marks the amino acid side chains involved in the formation of the active site (Figure 8A). 

Three amino acid side chains can be identified whose 3D structure can be aligned against each other, which are identical, and which are part of the respective active sites. An enclosed rectangle with a Roman numeral marks these amino acid side chains in the amino acid sequence in Figure 8A. The amino acid side chain marked by I corresponds to the GNE amino acid at position 476 and to the GlcNAc kinase amino acid at position 77, which was in both cases a glycine. The amino acid side chain marked with III was also a glycine, which was found at position 545 in the GNE and at position 128 in the GlcNAc kinase. Furthermore, this glycine is part of the ATP binding motifs DXGGT and GTG (see yellow rectangles in Figure 8A; [52,131]). The amino acid aspartic acid is located at position II in the sequence. This corresponds to amino acid position 517 in GNE and position 107 in GlcNAc kinase. 

The nearly conserved ATP binding motifs in both kinases appear to represent the greatest structural similarity. However, this is not a surprise since both require ATP to transfer the phosphate group to their respective substrate. 

The functions, pathogenicity predictions based on polymorphism phenotyping (PolyPhen), statements concerning disease association, genetic locations, and the database of single polymorphisms (dbSNP) IDs [132] of the amino acids at the positions I to III can be found in the table displayed in Figure 8C (everything, except for the function, was based on [133]).

## 4. Discussion and Outlook

All proteins/protein domains of interest—the GNE-epimerase domain, the GNE-kinase domain, and the GlcNAc kinase—could be successfully expressed in *E. coli*, purified via their 6xHis-tag, and post-translational modified by the two glycation agents methylglyoxal (MGO) and glyoxal (GO). Afterwards, different activity assays were performed to investigate the effect of glycation on the activity of the enzymes. In addition, we also examined the expression of GNE and the GlcNAc-kinase in undifferentiated, MGO/GO-treated C2C12 cells, a murine skeletal muscle cell line. 

The choice of a His-tag needs to be discussed, since it seems also possible that under certain conditions histidine side chains could be modified [134,135]. However, it seems that the histidine modifications described in these studies are based on oxidation and not on glycation, and require at least certain amounts of Cu(II) [135], so a His-tag seems like a safe choice from this standpoint. Furthermore, such a tag has already been used successfully in another study in connection with GNE [53]. However, the His-tag itself could also have an impact on the protein and its activity, e.g., due to steric hindrances or due to electrostatic interferences that result in impeded binding of the substrate to the active site [136,137]. Still, one could try introducing removable tags, such as the GST-tag (Glutathione *S*-Transferase-tag; [138], and see if the purification success stays the same or maybe even improves. Then, His-tag-based influences [139] could be excluded. 

The stronger CML signal when examining the GO-treated GNE domains as opposed to the MGO-treated ones can be explained by the fact that a fairly favorable pathway for the formation of CMLs is the reaction between lysine side chains and GO (see Figure 3A; [95,124]). In contrast, the reaction between lysine side chains and MGO leads to the formation of carboxyethyllysines (CELs) (indicated in blue in Figure 3A; [95,109]). It is therefore quite interesting that a putative specific antibody against carboxymethyllysines (CMLs) is also able to detect MGO-derived modifications on our proteins/protein domains of interest (see Figure 4). However, there are already some publications that document cross-reactions of anti-CML antibodies with CELs [140], respectively, with MGO-derived AGEs [86,141]. Further, it seems quite surprising that although the GNE-epimerase domain has almost twice as many lysines as the GNE-kinase domain, the relative glycation normalized to Ponceau of the kinase domain is more than twice as high compared to the epimerase domain and not the other way around. This could perhaps be a first indication of a higher responsiveness/vulnerability of the kinase to glycation. 

Based on the elevated MGO/GO-levels in diabetic patients [142,143], it might be interesting to investigate whether there are patients who suffer from both diseases—GNEM and diabetes mellitus (type I) and whether these patients develop the disease earlier (statistically) than patients suffering from GNEM alone.

One-step further, one could also split up all so far known mutations, into the groups:

1. Mutation introduces a new potential glycation site,
2. Mutation has no effect on the number of potential glycation sites (both amino acids lead to a potential glycation site),
3. Mutation deletes a potential glycation site.

This could then be used, for example, to examine whether different MGO/GO blood levels can be detected in the individual groups. 

Our preliminary analysis of the mutations listed on UniProt revealed 19 mutations that lead to a reduction in a potential glycation site (see Supplementary Table S18A). These mutations were mainly found in the epimerase domain and 31% of them are predicted to be benign. In addition, five mutations were found in which one amino acid causing a potential glycation site was exchanged for another, resulting in a new potential glycation site (see Supplementary Table S18B). Interestingly, in all five cases, an arginine was exchanged for a cysteine. Again, these mutations were predominantly found in the epimerase domain and are mostly damaging or pathogenic; the one found in the kinase domain was probably benign. Ten mutations were found that added an additional potential glycation site, 20% in the epimerase domain and 80% in the kinase domain. These mutations were mainly malignant (70%), 20% uncertain, and 10% benign. This may lead one to hypothesize that adding a potential glycation site is more likely to result in a malignant mutation—greater impact on the protein—than removing a potential glycation site. However, this needs to be verified further, e.g., in in vitro experiments on mutated versions of the protein. In addition, the conclusions/hypotheses of this preliminary mutation analysis can be linked to the other results in this study. Therefore, it seems fitting that adding an extra glycation site would have such a big impact on the kinase domain, which we have previously shown to be more responsive to glycation than the epimerase domain. 

Furthermore, there are genetic mutations within one family that result in different individual risks for disease progression and onset [12,103,144]. Since no genetic differences can be associated with the interindividual variability in the development of the disease, a possible difference in their lifestyles including different/other exposure of GNE to glycation agents can be considered. This could possibly explain the very different courses of the different mutations. 

It is already known that mutations in the *GNE* gene lead to the development of GNEM. Besides the introduction/deletion of glycation sites, mutations can also lead to changes at different structural levels of the protein [50,52] or to alterations in the interaction of substrate and protein [52]. Our experiments should help to see whether age might also affect the onset and progression of GNEM. 

**Figure 9:**
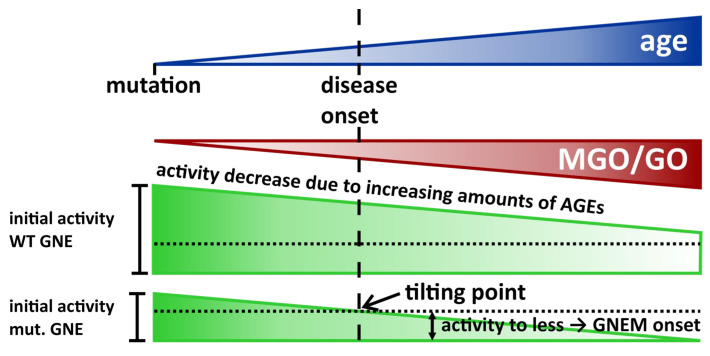
Schematic explaining the main hypothesis of the article—how age can influence the late onset of GNEM.

Based on our experiments, which showed reduced activities for the glycated GNEM, one could hypothesize that glycation, in addition to the effects that mutations already have on the enzyme activity [50,51], causes the activity to drop to such a level that it can no longer be compensated for (Figure 9). This hypothesis assumes a mutated *GNE* gene causes a mutated GNE enzyme that appears to work adequately for a certain period of time/the activity of the enzyme is sufficient for this period. Further, we would assume a constant decrease in activity, which is due to an increasing accumulation of PTMs on the enzyme, therefore assuming that these PTMs would also have a negative effect on the activity of the enzyme (proven in this study). This would lead to reaching and exceeding the tilting point, which in turn leads to the onset of the disease. 

In the case of one aforementioned diabetes mellitus and GNEM patient, the higher MGO/GO levels could increase the likelihood of reaching the required number of PTMs faster; scheme in Figure 9: the slope of the “activity triangle” increases, causing the tilting point to shift further to the left (Figure 9; towards younger years).

Nevertheless, the whole hypothesis has yet to be verified, for example, by comparing the activity of mutated enzymes with the activity of mutated and glycated enzymes, or by a study that initially deals with whether a glycated GNE can be detected at all—in cell culture and in vivo—to rule out that it is just an artefact that does not exist in reality. 

Only the individual domains of the GNE were examined in this study because the purification of the GNE as whole is still quite challenging. One critical point seems to be the removal of chaperone proteins [145]. Furthermore, our approach enables us to better estimate the individual effects of glycation of the two domains of the GNE. The overall effect of glycation on the protein still stays unknown, and it is also uncertain whether all the glycation sites that can be glycated when treated individually, are also accessible in the overall protein in such a way that glycation is also possible at these sites. What remains significant, however, is the fact that glycation of the individual domains leads to reduced enzyme activities; the effect on the GNE-kinase domain seems to be stronger than on the GNE-epimerase domain. In a next step, these experiments could be repeated on the overall protein.

Further, it would also be interesting to investigate the downstream effects of a glycated GNE in subsequent cell culture experiments. For example, one could check whether influences in sialylation are detectable. The result could then be compared with the assumption that hyposialylation play a role in GNEMs pathophysiology [16,17,18].

Furthermore, PTMs such as phosphorylation or *O*-GlcNAcylation were not considered here, although it has already been shown that these can also have an effect on activity [85,86]. However, *O*-GlcNAcylation and phosphorylation represent a special type of PTMs; they are enzymatic modifications [146,147]. Therefore, at least in the part of the study where only the protein was treated with the glycation agent, we exclude an influence of these types of PTMs on the activity of the GNE, since the enzymes required for them are missing.

In addition, however, it is quite possible that the glycation reagents react with the amino acid side chains of the protein in a different way than previously described in this study (see, e.g., Figure 3A), thereby generating a different type of PTM. As an example, lysine side chains could also be acylated by α-dicarbonyls as GO leading, e.g., to *N*^6^-glycoloyllysine (GALA; [148,149]). The extent to which acylation occurs in our proteins/protein domains of interest and what impact acylation can have on protein activity needs to be investigated in future studies.

Another interesting point was that the activity of the GlcNAc kinase, which, at least from just looking at its reaction schemes, appears to have the potential to stand in for the GNE kinase when needed, was not affected by GO or MGO and so seems to be resistant towards glycation—at least at the concentrations tested. 

However, since there are amino acids in the sequence of the GlcNAc kinase that can be glycated (see Figure 2Ca and Table 1) as well as structural similarities to the GNE kinase, other reasons must be found why this kinase appears to be resistant to glycation—under the given conditions. The different glycation resistance found in the two kinases could also just be an artefact due to the individual examination of the GNE kinase domain. Thus, it is possible that the GNE kinase exhibits the same glycation resistance as the GlcNAc kinase when examined as whole enzyme, together with the epimerase domain. A possible steric shielding of glycation sites by the epimerase domain could be a reason for this. All of this shows once again how important it would be to study the protein as a whole.

Another cause for the different behavior of the GlcNAc kinase could be that the investigated substrate ManNAc is not its main substrate [40,42]. It was already shown that the choice and availability of the substrate have an influence on the GlcNAc kinase activity, so only GlcNAc protects the GlcNAc kinase against cysteine modifiers and not ManNAc (MalNEt + GlcNAc: 70% ± 4% (remaining activity) whereas MalNEt + ManNAc: 2% ± 1% (remaining activity); [42]). This suggests that the binding and stabilization of ManNAc in the active site of the GlcNAc kinase differs from GlcNAc, and maybe the interaction partners/amino acids involved are less sensitive towards glycation. Therefore, it might be interesting to repeat the GlcNAc activity assay with GlcNAc as substrate and MGO/GO as the glycation agent so that one can compare the enzyme activity found there with the activity found with ManNAc as substrate. 

In the case that the GlcNAc kinase also shows a resistance to glycation with GlcNAc as substrate, it might be interesting to see how this might help in the treatment of GNEM. However, the biggest problem with seeing GlcNAc kinase as a possible replacement/substitute for the GNE-kinase is that the protein is not expressed in human skeletal muscle [150], which would make it impossible to function as a bypass in what are likely to be the most affected cells. On the other hand, reactivating GlcNAc kinase protein expression could be an interesting target in obtaining a functioning bypass for a non-/not-sufficient-working GNE-kinase domain. The RNA is at least expressed in the corresponding muscle cells [150,151]. Having said that, one should not disregard that GlcNAc kinase expression seems to play a role in certain types of human cancer [152].

Furthermore, it might also be interesting to investigate whether glycation can be reversed and thus the activity of the enzyme can be restored or at least increased back to a certain level (the pre-onset level). Fructosamine-3-kinase (FN3K; [153]) is such a protein that is able to de-stabilize fructosamines—glycation products, based on the reaction between glucose and an amino group—through phosphorylation and thus ultimately leads to their cleavage from the protein [154]. Therefore, it would be interesting to see if a correlation between FN3K and GNEM could be found—as it exists, for example, in relation to diabetes mellitus [155,156]. A recent study investigating the effect of ex vivo intravitreal FN3K injections on AGE-based cataracts seems to be quite promising as a potential new treatment of certain types of cataracts [157]. Therefore, it might be good to see if glucose-derived glycation products play a role in GNEM and whether activating the FN3K in muscle cells—maybe it might be possible to inject FN3K-solutions directly into the muscle (i.m.)—would remove these glycations, and so would raise the activity of the GNE back to the pre-onset level. 

Based on the aim to reduce or prevent glycations, one could also perform a correlation analysis of enzymes of the glyoxalase system and GNEM. The glyoxalase system is a system that can convert MGO into D-lactic acid [158,159]. Therefore, maybe upregulations of glyoxalase I could help to prevent the GNE from being glycated by MGO, which is a quite ubiquitous molecule in the human body, be it through ingestion through diet, or as byproduct of glycolysis, amino acid, and fatty acid degradation [114,115,116,158]. However, higher expression of glyoxalase I seems to be correlated with psychiatric problems such as anxiety [160,161], something to keep in mind, if such a study should ever be tested as a GNEM treatment. 

Altogether, our study showed that glycations with the glycation agents MGO and GO have a negative effect on the activity of the GNE-epimerase and GNE-kinase domain, but not on the GlcNAc kinase. Furthermore, GlcNAc kinase expression can be altered by GO in C2C12 cells, but not by MGO. However, none of the glycation agents tested altered the expression of the GNE. 

## Data Availability

All data can be found in this article and its supplement material.

## Supplementary Materials

The following supporting information can be downloaded at: https://www.mdpi.com/article/10.3390/biom13030422/s1; Supplementary Table S1: Anti-CML Western blot-band intensities normalized to Ponceau; Supplementary Figure S1: Western blots—GNE-epimerase domain; Supplementary Figure S2: Western blots—GNE-kinase domain; Supplementary Figure S3: Western blots—GlcNAc Kinase; Supplementary Table S2: Data—UDP-*N*-acetyl-glucosamine 2-epimerase activity assay; Supplementary Table S3: Two-Way analysis of variance (ANOVA) of the UDP-*N*-acetylglucosamine 2-epimerase activity assay; Supplementary Table S4: Tukey post hoc-test of the UDP-*N*-acetyl-glucosamine 2-epimerase activity assay; Supplementary Table S5: Student’s *t*-test of the UDP-*N*-acetylglucosamine 2-epimerase activity assay; Supplementary Table S6: Data—*N*-acetylmannosamine kinase activity assay; Supplementary Table S7: Two-Way analysis of variance (ANOVA) of the *N*-acetylmannosamine kinase activity assay; Supplementary Table S8: Tukey post hoc-test of the *N*-acetylmannosamine kinase activity assay—NADH consumption; Supplementary Table S9: Student’s *t*-test of the *N*-acetylmannosamine kinase activity assay—NADH consumption; Supplementary Table S10: *N*-acetylglucosamine kinase activity assay—NADH consumption; Supplementary Table S11: Two-Way analysis of variance (ANOVA) of the *N*-acetylglucosamine kinase activity assay; Supplementary Table S12: Tukey post hoc-test of the *N*-acetylglucosamine kinase activity assay—NADH consumption; Supplementary Table S13: Student’s *t*-test of the *N*-acetylglucosamine kinase activity assay—NADH consumption; Supplementary Figure S4: Unprocessed C2C12 cell culture images; Supplementary Figure S5: Western Blots C2C12 cell culture; Supplementary Table S14: Results from the MTT-Assay; Supplementary Table S15: *p*-values MTT-Assay; Supplementary Table S16: Results from the qPCR; Supplementary Table S17: *p*-values qPCR—GlcNAc kinase and GNE; Supplementary Table S18: UniProt-based analysis of mutations and their effect on potential glycation sites.
