# Supplementary material for: Glycation Interferes with the Activity of the Bi-Functional UDP-N-Acetylglucosamine 2-Epimerase/N-Acetyl-mannosamine Kinase (GNE)
Source: Biomolecules. 2023 Feb 23;13(3):422. doi: 10.3390/biom13030422 (PMC10046061; doi:10.3390/biom13030422)
Supplement: Supplementary file 1 [file biomolecules-13-00422-s001.zip › biomolecules-2149063-supplementary.pdf]

Supplementary material of the article

Glycation interferes with the activity of the bi-functional UDP-*N*-acetylglucosamine 2 -epimerase/*N*-acetylmannosamine kinase (GNE)

Vanessa Hagenhaus[1], Jacob L. Gorenflos López[2,3], Rebecca Rosenstengel[1], Carolin Neu[1], Christian P. R. Hackenberger[2,3], Arif Celik[2,3], Klara Weinert[1], Mai-Binh Nguyen[1], Kaya Bork[1], Rüdiger Horstkorte[1], and Astrid Gesper[1]*

1. Institute for Physiological Chemistry, Medical Faculty, Martin-Luther-University Halle-Wittenberg, 06114 Halle (Saale), Germany

2. Leibniz-Forschungsinstitut für Molekulare Pharmakologie im Forschungsverbund Berlin e.V. (FMP), Campus Berlin-Buch, Robert-Roessle-Str. 10, 13125 Berlin, Germany

3. Humboldt Universität zu Berlin, Institut für Chemie, Brook-Taylor-Str. 2, 12489 Berlin, Germany

Supplementary Table S1:

Anti-CML Western blot-band intensities normalized to Ponceau:

UDP-*N*-acetylglucosamine 2- epimerase				
**MGO**	**0 mM MGO**	**0.5 mM MGO**	**2 mM MGO**	**5 mM MGO**
mean/b.i.	1.00 b.i.	4.64 b.i.	10.58 b.i.	25.77 b.i.
standard deviation/b.i.	0.00 b.i.	1.43 b.i.	6.23 b.i.	15.18 b.i.
**GO**	**0 mM GO**	**0.5 mM GO**	**2 mM GO**	**5 mM GO**
mean/b.i.	1.00 b.i.	22.29 b.i.	79.86 b.i.	170.89 b.i.
standard deviation/b.i.	0.00 b.i.	16.93 b.i.	23.33 b.i.	89.16 b.i.
***N*-acetylmannosamine kinase**				
**MGO**	**0 mM MGO**	**0.5 mM MGO**	**2 mM MGO**	**5 mM MGO**
mean/b.i.	1.00 b.i.	4.23 b.i.	72.89 b.i.	165.25 b.i.
standard deviation/b.i.	0.00 b.i.	2.03 b.i.	56.82 b.i.	128.03 b.i.
**GO**	**0 mM GO**	**0.5 mM GO**	**2 mM GO**	**5 mM GO**
mean/b.i.	1.00 b.i.	66.18 b.i.	282.48 b.i.	518.37 b.i.
standard deviation/b.i.	0.00 b.i.	6.78 b.i.	57.10 b.i.	51.41 b.i.
***N*-acetylglucosamine kinase**				
**MGO**	**0 mM MGO**	**0.5 mM MGO**	**2 mM MGO**	**5 mM MGO**
mean/b.i.	1.00 b.i.	14.11 b.i.	53.23 b.i.	95.25 b.i.
standard deviation/b.i.	0.00 b.i.	5.65 b.i.	23.84 b.i.	82.21 b.i.
**GO**	**0 mM GO**	**0.5 mM GO**	**2 mM GO**	**5 mM GO**
mean/b.i.	1.00 b.i.	1.90 b.i.	16.25 b.i.	94.76 b.i.
standard deviation/b.i.	0.00 b.i.	0.18 b.i.	3.50 b.i.	13.68 b.i.

Supplementary Figure S1:

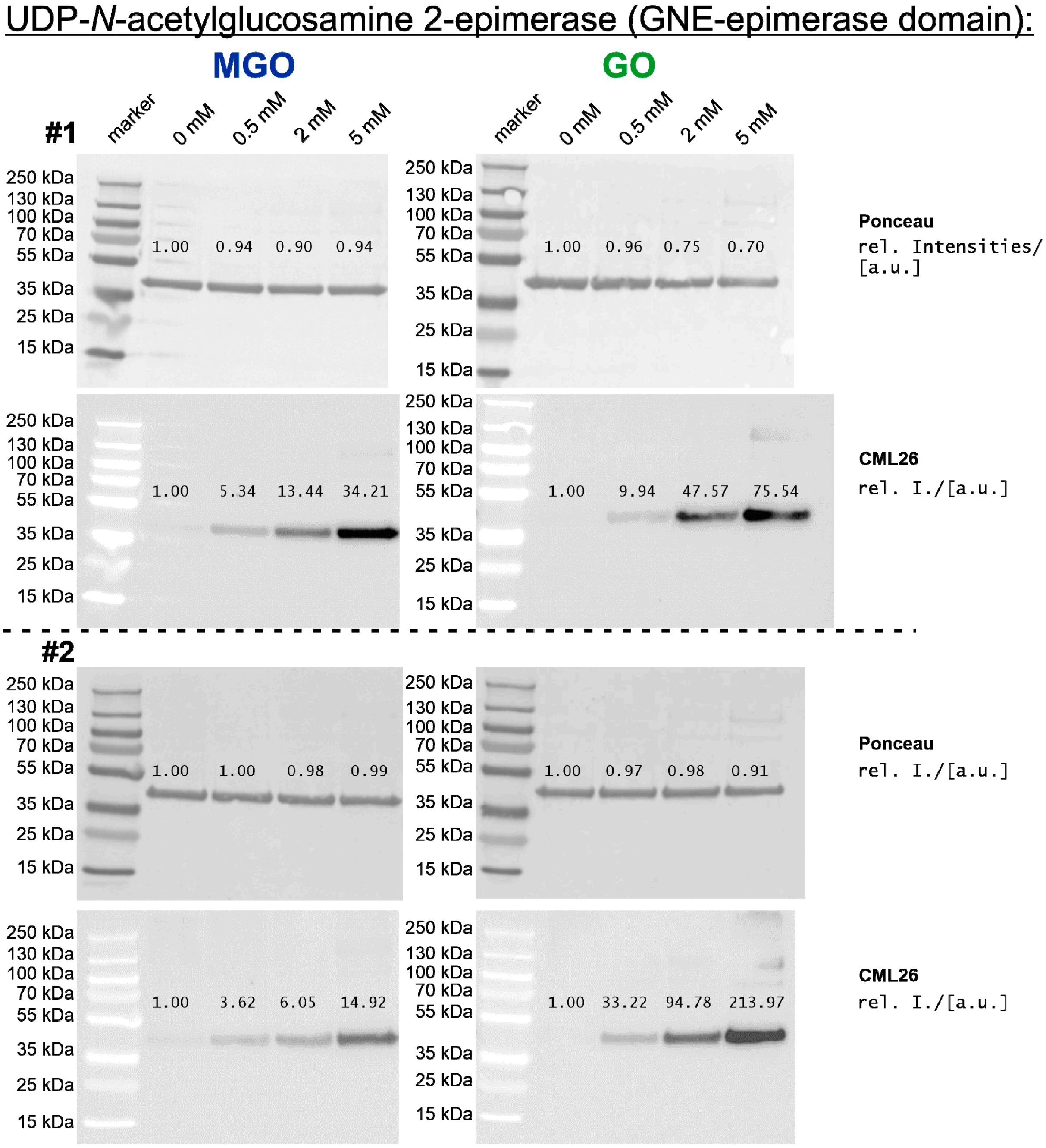

Supplementary Figure S2:

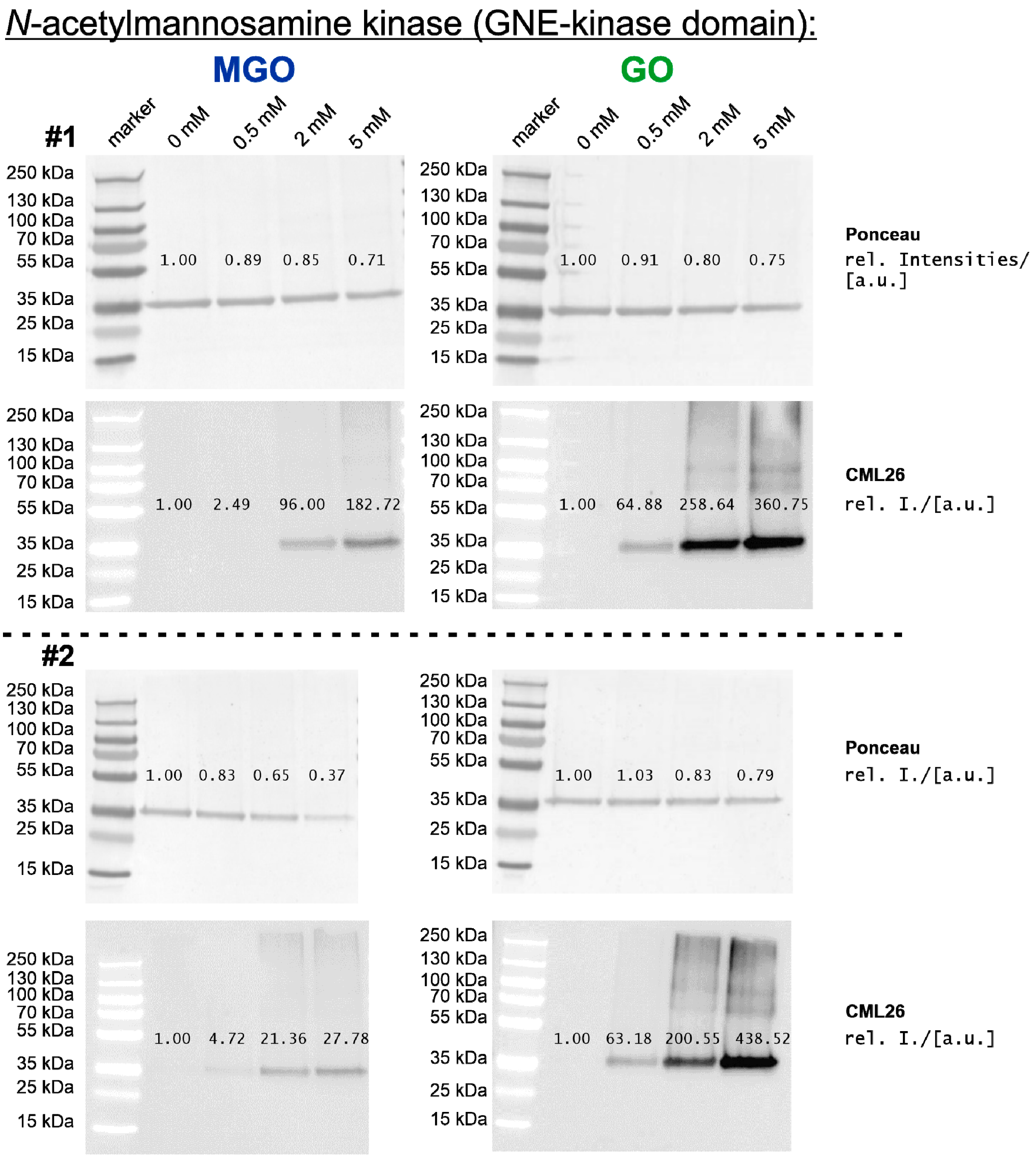

Supplementary Figure S3:

N-acetylglucosamine kinase (GlcNAc kinase):

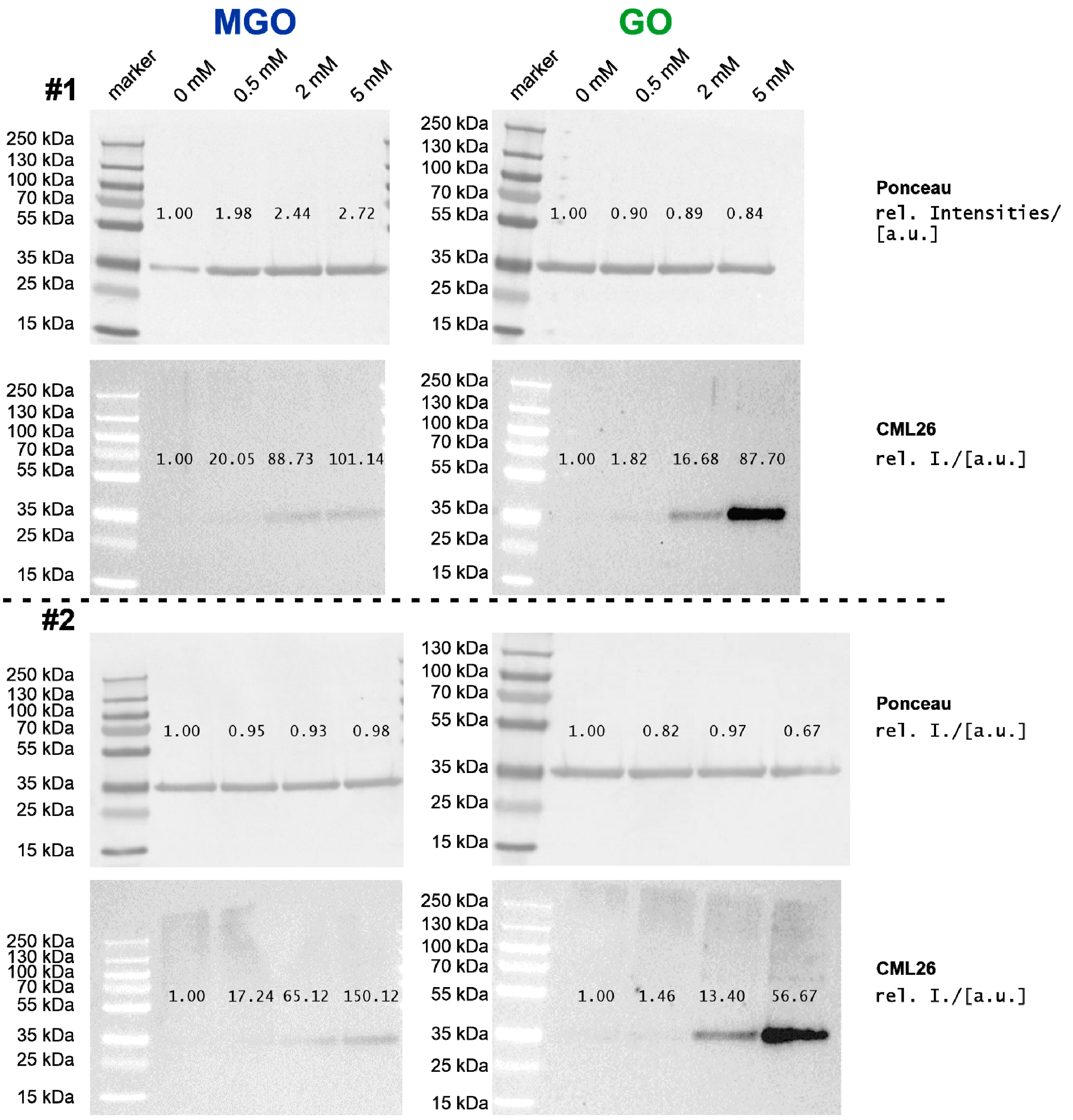

Supplementary Table S2:

UDP-*N*-acetylglucosamine 2-epimerase activity assay:

UDP-Konzentration	neg. ctrl.	pos. ctrl.	0.5 mM MGO	2 mM MGO	5 mM MGO	0.5 mM GO	2 mM GO	5 mM GO
mean/nM	26.19 nM	169.49 nM	142.88 nM	125.95 nM	102.19 nM	120.60 nM	109.63 nM	106.71 nM
median/nM	17.26 nM	158.98 nM	126.48 nM	123.85 nM	107.55 nM	119.32 nM	107.93 nM	106.44 nM
standard deviation/ nM	15.66 nM	44.16 nM	27.33 nM	16.17 nM	13.37 nM	11.88 nM	7.22 nM	8.65 nM
standard error/ nM	7.00 nM	19.75 nM	12.22 nM	7.23 nM	5.98 nM	5.31 nM	3.23 nM	3.87 nM
minimum/nM	13.34 nM	127.75 nM	119.90 nM	111.39 nM	86.33 nM	103.99 nM	103.66 nM	95.72 nM
maximum/nM	47.15 nM	237.90 nM	173.36 nM	150.10 nM	118.24 nM	136.84 nM	121.35 nM	118.67 nM
interquartile range (IQR)/nM	24.35 nM	47.68 nM	49.44 nM	20.90 nM	17.98 nM	6.64 nM	6.95 nM	8.41 nM

Supplementary Table S3:

Two-Way analysis of variance (ANOVA) of the UDP-*N*-acetylglucosamine 2-epimerase activity assay:

factor	*p*-value	significance asterisks
concentration	1.43E-03	***
glycation substance	1.96E-01	-
concentration*glycation substance	3.58E-03	***

Supplementary Table S4:

Tukey *post hoc*-test of the UDP-*N*-acetylglucosamine 2-epimerase activity assay:

condition A	condition B	*p*-value	significance asterisks
**0.5 mM MGO**	**2 mM MGO**	2.22E-01	-
**0.5 mM MGO**	**5 mM MGO**	5.86E-03	**
**2 mM MGO**	**5 mM MGO**	7.76E-02	-
**0.5 mM GO**	**2 mM GO**	4.95E-01	-
**0.5 mM GO**	**5 mM GO**	3.42E-01	-
**2 mM GO**	**5 mM GO**	9.47E-01	-
**MGO**	**GO**	1.01E-01	-
**0.5 mM**	**2 mM**	1.46E-01	-
**0.5 mM**	**5 mM**	7.91E-03	**
**2 mM**	**5 mM**	1.67E-01	-
**0.5 mM MGO**	**0.5 mM GO**	4.32E-02	*
**2 mM MGO**	**2 mM GO**	1.17E-01	-
**5 mM MGO**	**5 mM GO**	6.40E-01	-

Supplementary Table S5:

Student's *t*-test of the UDP-*N*-acetylglucosamine 2-epimerase activity assay:

condition A	condition B	*p*-value	significance asterisks
**positive control**	**negative control**	3.47E-03	***
**positive control**	**0.5 mM MGO**	1.77E-01	-
**positive control**	**2 mM MGO**	3.19E-02	*
**positive control**	**5 mM MGO**	1.70E-02	*
**positive control**	**0.5 mM GO**	3.34E-02	*
**positive control**	**2 mM GO**	4.46E-02	*
**positive control**	**5 mM GO**	4.03E-02	*
**negative control**	**0.5 mM MGO**	2.49E-03	***
**negative control**	**2 mM MGO**	1.23E-03	***
**negative control**	**5 mM MGO**	4.05E-03	***
**negative control**	**0.5 mM GO**	3.90E-04	***
**negative control**	**2 mM GO**	1.51E-04	***
**negative control**	**5 mM GO**	3.79E-04	***

Supplementary Table S6:

N-acetylmannosamine kinase activity assay – NADH consumption:

NADH-consumption	neg. ctrl.	pos. ctrl.	0.5 mM MGO	2 mM MGO	5 mM MGO	0.5 mM GO	2 mM GO	5 mM GO
mean/fmol·min^{-}	29.62 fmol·min^{-1}	556.97 fmol·min^{-}	407.03 fmol·min^{-1}	169.69 fmol·min^{-1}	74.97 fmol·min^{-1}	495.50 fmol·min^{-1}	274.65 fmol·min^{-1}	122.51 fmol·min^{-}
median/fmol·min^{-}	18.29 fmol·min^{-1}	557.70 fmol·min^{-}	411.42 fmol·min^{-}	170.05 fmol·min^{-1}	73.14 fmol·min^{-1}	522.96 fmol·min^{-1}	274.28 fmol·min^{-1}	115.20 fmol·min^{-1}
standard deviation/ fmol·min^{-1}	26.61 fmol·min^{-1}	43.07 fmol·min^{-1}	14.94 fmol·min^{-}	42.46 fmol·min^{-1}	28.74 fmol·min^{-1}	52.51 fmol·min^{-1}	27.35 fmol·min^{-1}	22.09 fmol·min^{-1}
standard error/ fmol·min^{-1}	11.90 fmol·min^{-}	19.26 fmol·min^{-1}	6.68 fmol·min^{-1}	18.99 fmol·min^{-1}	12.85 fmol·min^{-1}	23.48 fmol·min^{-1}	12.23 fmol·min^{-1}	9.88 fmol·min^{-}
minimum/fmol·min^{-1}	5.49 fmol·min^{-}	508.33 fmol·min^{-}	389.48 fmol·min^{-1}	113.37 fmol·min^{-1}	32.91 fmol·min^{-1}	427.88 fmol·min^{-1}	241.37 fmol·min^{-1}	100.57 fmol·min^{-}
maximum/fmol·min^{-}	65.83 fmol·min^{-1}	614.39 fmol·min^{-}	422.39 fmol·min^{-1}	217.60 fmol·min^{-1}	106.06 fmol·min^{-}	544.90 fmol·min^{-1}	312.68 fmol·min^{-1}	155.43 fmol·min^{-}
interquartile range (IQR)/fmol·min^{-}	40.23 fmol·min^{-1}	58.51 fmol·min^{-1}	25.60 fmol·min^{-}	58.51 fmol·min^{-1}	31.09 fmol·min^{-1}	80.46 fmol·min^{-1}	29.26 fmol·min^{-1}	25.60 fmol·min^{-1}

Supplementary Table S7:

Two-Way analysis of variance (ANOVA) of the *N*-acetylmannosamine kinase activity assay:

factor	p-value	significance asterisks
concentration	4.11E-04	***
glycation substance	2.42E-03	***
concentration*glycation substance	1.22E-01	-

Supplementary Table S8:

Tukey *post hoc*-test of the *N*-acetylmannosamine kinase activity assay – NADH consumption:

condition A	condition B	*p*-value	significance asterisks
**0.5 mM MGO**	**2 mM MGO**	4.27E-06	***
**0.5 mM MGO**	**5 mM MGO**	2.72E-07	***
**2 mM MGO**	**5 mM MGO**	2.82E-03	***
**0.5 mM GO**	**2 mM GO**	7.31E-06	***
**0.5 mM GO**	**5 mM GO**	7.67E-08	***
**2 mM GO**	**5 mM GO**	1.18E-04	***
**MGO**	**GO**	1.64E-03	***
**0.5 mM**	**2 mM**	3.38E-07	***
**0.5 mM**	**5 mM**	0	***
**2 mM**	**5 mM**	4.33E-05	***
**0.5 mM MGO**	**0.5 mM GO**	1.64E-03	***
**2 mM MGO**	**2 mM GO**	5.79E-04	***
**5 mM MGO**	**5 mM GO**	3.76E-02	*

Supplementary Table S9:

Student's *t*-test of the *N*-acetylmannosamine kinase activity assay – NADH consumption:

condition A	condition B	*p*-value	significance asterisks
**positive control**	**negative control**	1.40E-02	*
**positive control**	**0.5 mM MGO**	1.32E-03	***
**positive control**	**2 mM MGO**	8.23E-06	***
**positive control**	**5 mM MGO**	8.23E-06	***
**positive control**	**0.5 mM GO**	1.19E-01	-
**positive control**	**2 mM GO**	1.80E-04	***
**positive control**	**5 mM GO**	3.46E-06	***
**negative control**	**0.5 mM MGO**	3.30E-02	*
**negative control**	**2 mM MGO**	1.82E-01	-
**negative control**	**5 mM MGO**	3.59E-01	-
**negative control**	**0.5 mM GO**	1.71E-02	*
**negative control**	**2 mM GO**	8.33E-02	-
**negative control**	**5 mM GO**	2.23E-01	-

Supplementary Table S10:

N-acetylglucosamine kinase activity assay – NADH consumption:

	neg. ctrl.	pos. ctrl.	0.5 mM MGO	2 mM MGO	5 mM MGO	0.5 mM GO	2 mM GO	5 mM GO
mean/fmol·min^{-1}	12.89 fmol·min^{-1}	93.35 fmol·min^{-1}	89.23 fmol·min^{-1}	79.08 fmol·min^{-1}	82.01 fmol·min^{-1}	88.87 fmol·min^{-1}	87.40 fmol·min^{-1}	89.69 fmol·min^{-1}
median/fmol·min^{-1}	16.91 fmol·min^{-1}	84.57 fmol·min^{-1}	87.31 fmol·min^{-1}	80.00 fmol·min^{-1}	80.91 fmol·min^{-1}	85.48 fmol·min^{-1}	79.54 fmol·min^{-1}	80.46 fmol·min^{-1}
standard deviation/ fmol·min^{-1}	9.95 fmol·min^{-1}	37.80 fmol·min^{-1}	5.10 fmol·min^{-1}	6.68 fmol·min^{-1}	5.24 fmol·min^{-1}	9.51 fmol·min^{-1}	12.30 fmol·min^{-1}	23.41 fmol·min^{-1}
standard error/ fmol·min^{-1}	4.45 fmol·min^{-1}	16.90 fmol·min^{-1}	2.28 fmol·min^{-1}	2.99 fmol·min^{-1}	2.34 fmol·min^{-1}	4.25 fmol·min^{-1}	5.50 fmol·min^{-1}	10.47 fmol·min^{-1}
minimum/fmol·min^{-1}	-2.29 fmol·min^{-1}	67.66 fmol·min^{-1}	83.66 fmol·min^{-1}	69.94 fmol·min^{-1}	76.80 fmol·min^{-1}	77.71 fmol·min^{-1}	76.34 fmol·min^{-1}	65.83 fmol·min^{-1}
maximum/fmol·min^{-1}	21.94 fmol·min^{-1}	159.08 fmol·min^{-1}	96.91 fmol·min^{-1}	85.94 fmol·min^{-1}	90.51 fmol·min^{-1}	100.11 fmol·min^{-1}	101.03 fmol·min^{-1}	127.54 fmol·min^{-1}
interquartile range (IQR)/fmol·min^{-1}	11.43 fmol·min^{-1}	18.29 fmol·min^{-1}	4.57 fmol·min^{-1}	9.60 fmol·min^{-1}	3.66 fmol·min^{-1}	13.71 fmol·min^{-1}	21.03 fmol·min^{-1}	13.71 fmol·min^{-1}

Supplementary Table S11:

<u>Two-Way analysis of variance (ANOVA) of the *N*-acetylglucosamine kinase activity assay:</u>

factor	p-value	significance asterisks
concentration	1.59E-01	-
glycation substance	2.98E-01	-
concentration*glycation substance	8.75E-02	-

Supplementary Table S12:

<u>Tukey *post hoc*-test of the *N*-acetylglucosamine kinase activity assay – NADH consumption:</u>

condition A	condition B	*p*-value	significance asterisks
**0.5 mM MGO**	**2 mM MGO**	4.20E-01	-
**0.5 mM MGO**	**5 mM MGO**	6.29E-01	-
**2 mM MGO**	**5 mM MGO**	9.23E-01	-
**0.5 mM GO**	**2 mM GO**	9.80E-01	-
**0.5 mM GO**	**5 mM GO**	9.94E-01	-
**2 mM GO**	**5 mM GO**	9.52E-01	-
**MGO**	**GO**	3.03E-01	-
**0.5 mM**	**2 mM**	5.55E-01	-
**0.5 mM**	**5 mM**	8.28E-01	-
**2 mM**	**5 mM**	8.81E-01	-
**0.5 mM MGO**	**0.5 mM GO**	9.63E-01	-
**2 mM MGO**	**2 mM GO**	3.08E-01	-
**5 mM MGO**	**5 mM GO**	3.44E-01	-

Supplementary Table S13:

Student's *t*-test of the *N*-acetylglucosamine kinase activity assay – NADH consumption:

condition A	condition B	*p*-value	significance asterisks
**positive control**	**negative control**	7.59E-03	**
**positive control**	**0.5 mM MGO**	8.32E-01	-
**positive control**	**2 mM MGO**	4.64E-01	-
**positive control**	**5 mM MGO**	5.43E-01	-
**positive control**	**0.5 mM GO**	8.33E-01	-
**positive control**	**2 mM GO**	7.84E-01	-
**positive control**	**5 mM GO**	7.47E-01	-
**negative control**	**0.5 mM MGO**	1.16E-04	***
**negative control**	**2 mM MGO**	6.81E-04	***
**negative control**	**5 mM MGO**	2.19E-04	***
**negative control**	**0.5 mM GO**	8.52E-04	***
**negative control**	**2 mM GO**	1.55E-03	***
**negative control**	**5 mM GO**	1.85E-03	***

Supplementary Figure S4:
Unprocessed C2C12 cell culture images.

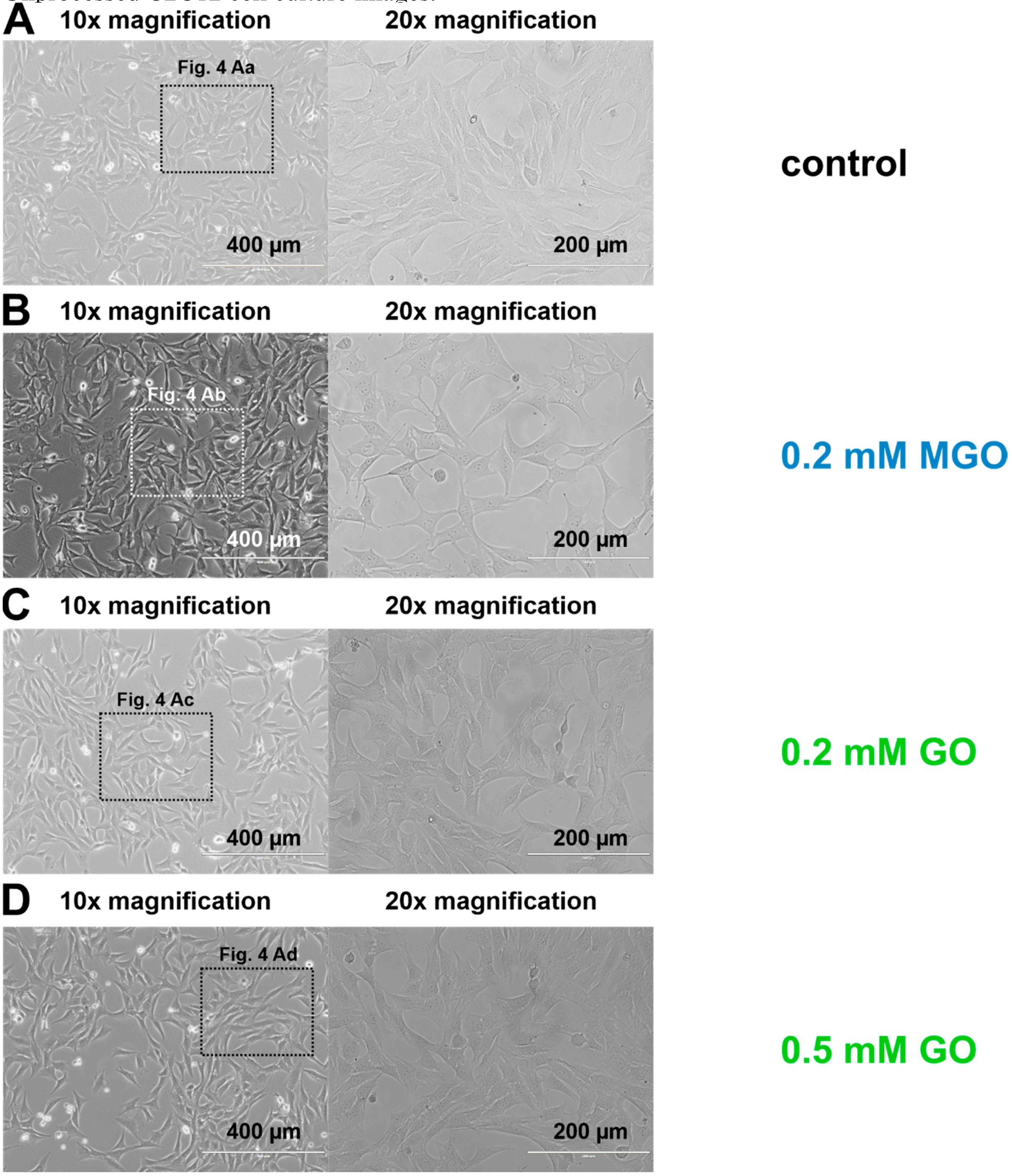

Supplementary Figure S5:
Western Blots C2C12-cell culture

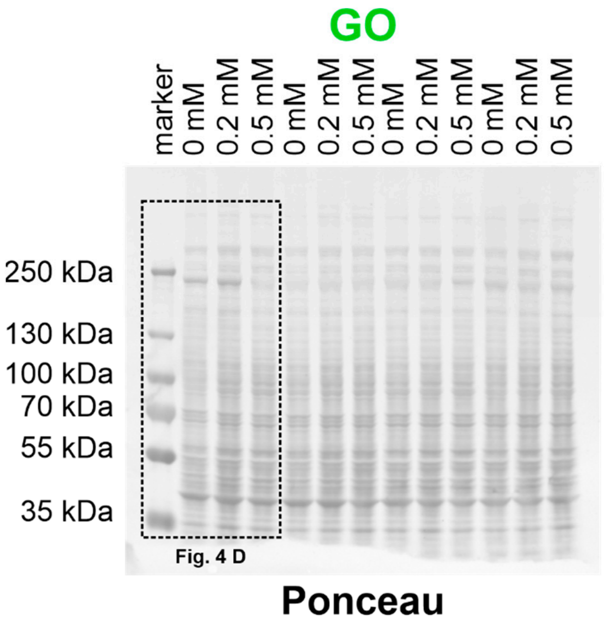

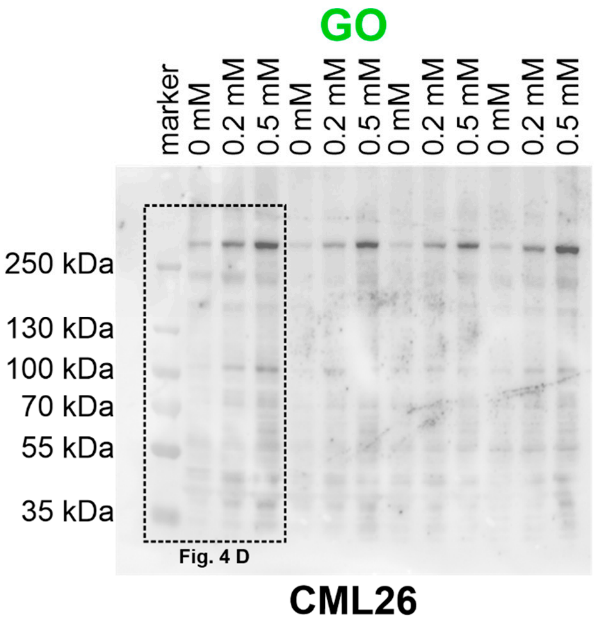

normalized to Ponceau (n=4):

	0 mM	0.2 mM	0.5 mM
mean/relative b.i.	1.00	1.38	2.10
standard deviation/ relative b.i.	0.00	0.11	0.54

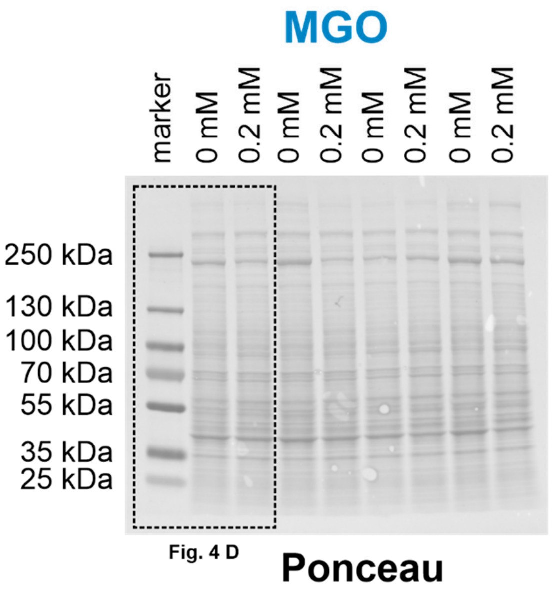

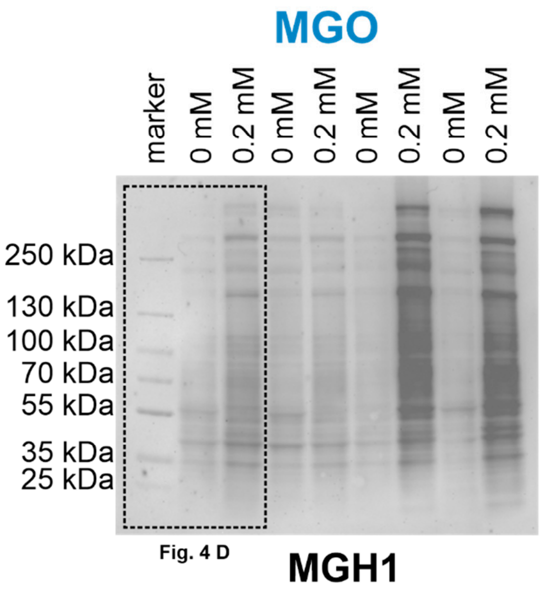

normalized to Ponceau (n=4):

	0 mM	0.2 mM
mean/relative b.i.	1.00	14.22
standard deviation/relative b.i.	0.00	14.71

Supplementary Table S14:
<u>Results from the MTT-Assay</u>

GO		
**concentration/mM**	**mean/a.u.**	**standard deviation/a.u.**
0 mM	1.00 a.u.	0.00 a.u.
0.5 mM	1.09 a.u.	0.09 a.u.
1 mM	0.90 a.u.	0.12 a.u.
1.5 mM	0.93 a.u.	0.09 a.u.
2 mM	0.83 a.u.	0.05 a.u.
2.5 mM	0.77 a.u.	0.01 a.u.
MGO		
**concentration/mM**	**mean/a.u.**	**standard deviation/a.u.**
0 mM	1.00 a.u.	0.00 a.u.
0.1 mM	1.10 a.u.	0.09 a.u.
0.2 mM	1.04 a.u.	0.08 a.u.
0.5 mM	0.85 a.u.	0.09 a.u.
0.75 mM	0.58 a.u.	0.06 a.u.
1 mM	0.32 a.u.	0.07 a.u.

Supplementary Table S15:
<u>*p*-values MTT-Assay</u>

condition A	condition B	*p*-value	significance asterisks
control	**0.5 mM GO**	3.10E-01	-
control	**1 mM GO**	3.60E-01	-
control	**1.5 mM GO**	3.84E-01	-
control	**2 mM GO**	3.37E-02	*
control	**2.5 mM GO**	6.00E-04	***
control	**0.1 mM MGO**	2,81E-01	-
control	**0.2 mM MGO**	5.37E-01	-
control	**0.5 mM MGO**	1.38E-01	-
control	**0.75 mM MGO**	1.07E-02	*
control	**1 mM MGO**	5.00E-03	***

Supplementary Table S16:

Results from the qPCR (Control and 0.5 mM GO):

Cq-value								
	Control				0.5 mM GO			
RPL 26	16.32	16.22	15.70	15.69	16.15	16.18	15.52	15.47
GAPDH	15.16	15.64	14.96	14.93	15.42	15.62	14.69	14.71
GNE Isoform 1	31.72	31.77	31.38	30.28	31.36	31.39	31.22	31.04
GNE Isoform 2	27.51	29.41	28.54	28.30	29.90	29.93	30.48	29.38
GlcNAcKinase	21.51	21.59	21.42	20.82	21.85	22.02	21.51	21.11
GNE	22.98	23.36	22.50	22.31	23.24	23.74	22.58	22.33
dCq-value								
RPL 26	-1.16	-0.57	-0.74	-0.75	-0.73	-0.56	-0.83	-0.76
GAPDH	15.40	15.55	15.68	14.59	15.21	15.21	15.70	15.57
GNE Isoform 1	11.19	13.20	12.84	12.62	13.75	13.76	14.96	13.91
GNE Isoform 2	5.19	5.38	5.72	5.13	5.70	5.84	5.99	5.64
GlcNAcKinase	6.66	7.14	6.80	6.63	7.09	7.56	7.06	6.86
GNE	-16.32	-16.22	-15.70	-15.69	-16.15	-16.18	-15.52	-15.47
ddCq-value								
RPL 26	0.00	0.00	0.00	0.00	0.00	0.00	0.00	0.00
GAPDH	-0.35	0.23	0.07	0.05	0.08	0.25	-0.03	0.05
GNE Isoform 1	0.09	0.25	0.38	-0.71	-0.10	-0.09	0.39	0.26
GNE Isoform 2	-1.27	0.74	0.38	0.16	1.29	1.30	2.50	1.45
GlcNAc Kinase	-0.17	0.02	0.36	-0.22	0.35	0.49	0.63	0.28
GNE	-0.15	0.34	-0.01	-0.18	0.29	0.75	0.25	0.05
FC (fold change)								
RPL 26	1.00	1.00	1.00	1.00	1.00	1.00	1.00	1.00
GAPDH	1.28	0.85	0.95	0.96	0.95	0.84	1.02	0.97
GNE Isoform 1	0.94	0.84	0.77	1.64	1.07	1.07	0.76	0.83
GNE Isoform 2	2.41	0.60	0.77	0.90	0.41	0.41	0.18	0.37
GlcNAc Kinase	1.12	0.98	0.78	1.16	0.78	0.71	0.65	0.82
GNE	1.11	0.79	1.00	1.13	0.82	0.59	0.84	0.97

Supplementary Table S17:

p-values qPCR – GlcNAc kinase:

condition A	condition B	*p*-value	significance asterisks
control	**0.2 mM MGO**	5.49E-02	-
control	**0.2 mM GO**	8.61E-01	-
control	**0.5 mM GO**	4.56E-02	*

p-values qPCR – GNE:

condition A	condition B	*p*-value	significance asterisks
control	**0.2 mM MGO**	1.32E-01	-
control	**0.2 mM GO**	6.41E-01	-
control	**0.5 mM GO**	1.11E-01	-

Supplementary Table S18:

UniProt-based analysis of mutations and their effect on potential glycation sites

https://www.uniprot.org – searching for: "Q9Y223" – 07.12.2022

We filtered the mutations based on following parameters: "likely disease" + "missense" + "not sialuria".

First column: **E:** Epimerase domain, or **K:** Kinase domain

Second Column: **S:** Sheet, **H:** Helix, or **L:** Loop;

Fifth Column: **Blue:** basic amino acid, **Red:** acidic amino acid, **Yellow:** polar amino acid, or **Green:** apolar amino acid.

(A)

Reduction of a potential glycation site

		dbSNP	Position	Change	PolyPhen-Score/Clinical significance
E	S	rs1401082364	11	R>Q	0.086 (benign)
E	S	rs769716748	11	R>W	0.73 (possibly damaging)
E	S	rs1209266607	13	C>S	0.262 (benign)
E	S	rs886044539	71	R>W	0.669 (possible damaging)
E	S	rs748704459	129	R>Q	0.985 (probably damaging)
E	H	rs1157107103	152	K>T	0.999 (probably damaging)
E	S	rs772597073	177	R>H	0.156 (benign)
E	S	rs121908629	246	R>Q	Likely pathogenic
E	S	rs773729410	246	R>W	Likely pathogenic
E	L	rs121908633	303	C>V	Likely pathogenic
E	L	rs1455785164	306	R>Q	Likely pathogenic
E	S	rs150132839	335	R>W	0.94 (probably damaging)
E	L	rs1554660090	353	K>E	0.916 (probably damaging)
E	H	rs1828924556	375	K>T	Variant of uncertain significance
E	L	rs1828920194	386	K>N	Variant of uncertain significance
E	L	rs1180382282	386	K>Q	0.003 (benign)
K	S	rs780092539	420	R>Q	0.957 (probably damaging)
K	S	rs138357804	481	R>Q	0.001 (benign)
K	H	rs139347806	684	R>S	0 (benign)

(B)

No change in the number of potential glycation sites

		dbSNP	Position	Change	PolyPhen-Score/Clinical significance
E	L	rs148523065	101	R>C	0.98 (probably damaging)
E	H	rs769215411	162	R>C	Likely pathogenic
E	S	rs539332585	177	R>C	Likely pathogenic
E	S	rs762106720	277	R>C	0.877 (possibly damaging)
K	H	rs139347806	684	R>C	0.118 (benign)

(C)

Addition of one potential glycation site

		dbSNP	Position	Change	PolyPhen-Score/Clinical significance
E	S	rs886043739	110	H>R	0.998 (probably damaging)
E	L	rs755743750	304	G>R	0.997 (probably damaging)
K	S	rs866841518	416	G>R	1 (probably damaging)
K	S	rs1313840587	423	I>R	possibly damaging (0.9)
K	S	rs1828703850	429	E>K	Variant of uncertain significance
K	H	rs1337925138	443	E>K	0.119 (benign)
K	L	rs986773986	528	F>C	Likely pathogenic
K	L	rs762009737	559	G>R	1 (probably damaging)
K	S	rs1828512489	577	P>R	Variant of uncertain significance
K	S	rs776384541	669	G>R	0.999 (probably damaging)
